# Using wearable devices to generate real-world, individual-level data in rural, low-resource contexts in Burkina Faso, Africa: A case study

**DOI:** 10.3389/fpubh.2022.972177

**Published:** 2022-09-30

**Authors:** Sophie Huhn, Ina Matzke, Mara Koch, Hanns-Christian Gunga, Martina Anna Maggioni, Ali Sié, Valentin Boudo, Windpanga Aristide Ouedraogo, Guillaume Compaoré, Aditi Bunker, Rainer Sauerborn, Till Bärnighausen, Sandra Barteit

**Affiliations:** ^1^Faculty of Medicine and University Hospital, Heidelberg Institute of Global Health (HIGH), Heidelberg University, Heidelberg, Germany; ^2^Charité – Universitätsmedizin Berlin, Institute of Physiology, Center for Space Medicine and Extreme Environments Berlin, Berlin, Germany; ^3^Department of Biomedical Sciences for Health, Università Degli Studi di Milano, Milano, Italy; ^4^Centre de Recherche en Santé, Nouna, Burkina Faso; ^5^Department of Global Health and Population, Harvard T.H. Chan School of Public Health, Boston, MA, United States; ^6^Africa Health Research Institute (AHRI), KwaZulu-Natal, South Africa

**Keywords:** wearables, consumer-based wearables, digital technologies, health research, real world data, SSA, sub-Saharan Africa, global health

## Abstract

**Background:**

Wearable devices may generate valuable data for global health research for low- and middle-income countries (LMICs). However, wearable studies in LMICs are scarce. This study aims to investigate the use of consumer-grade wearables to generate individual-level data in vulnerable populations in LMICs, focusing on the acceptability (quality of the devices being accepted or even liked) and feasibility (the state of being workable, realizable, and practical, including aspects of data completeness and plausibility).

**Methods:**

We utilized a mixed-methods approach within the health and demographic surveillance system (HDSS) to conduct a case study in Nouna, Burkina Faso (BF). All HDSS residents older than 6 years were eligible. *N* = 150 participants were randomly selected from the HDSS database to wear a wristband tracker (Withings Pulse HR) and *n* = 69 also a thermometer patch (Tucky thermometer) for 3 weeks. Every 4 days, a trained field worker conducted an acceptability questionnaire with participants, which included questions for the field workers as well. Descriptive and qualitative thematic analyses were used to analyze the responses of study participants and field workers.

**Results:**

In total, *n* = 148 participants were included (and n = 9 field workers). Participant's acceptability ranged from 94 to 100% throughout the questionnaire. In 95% of the cases (*n* = 140), participants reported no challenges with the wearable. Most participants were not affected by the wearable in their daily activities (*n* = 122, 83%) and even enjoyed wearing them (*n* = 30, 20%). Some were concerned about damage to the wearables (*n* = 7, 5%). Total data coverage (i.e., the proportion of the whole 3-week study duration covered by data) was 43% for accelerometer (activity), 3% for heart rate, and 4% for body shell temperature. Field workers reported technical issues like faulty synchronization (*n* = 6, 1%). On average, participants slept 7 h (SD 3.2 h) and walked 8,000 steps per day (SD 5573.6 steps). Acceptability and data completeness were comparable across sex, age, and study arms.

**Conclusion:**

Wearable devices were well-accepted and were able to produce continuous measurements, highlighting the potential for wearables to generate large datasets in LMICs. Challenges constituted data missingness mainly of technical nature. To our knowledge, this is the first study to use consumer-focused wearables to generate objective datasets in rural BF.

## Introduction

### Wearables for health research in low- and middle-income countries

Wearable devices increasingly find their way into health care and health research (1). For example, advantages of consumer-based wearables compared to research-grade devices and questionnaires are low costs, user-friendliness, and unobtrusiveness, as well as the ability to collect data in the natural environment of study participants (2). Also, the accuracy and reliability of these devices have improved, even leading to clinically approved certifications [like the US Food and Drug Approval or the European CE approval (2, 3)].

Wearable data thus may generate valuable data for global health research even on an individual level (4), as wearables allow for remote measurements of continuous physiological data in the wearers natural environment (so-called ecological momentary assessment) (2).

Wearables are already used to forecast infectious disease outbreaks (5, 6) and gain population-based insights using big data (2, 7) or conduct healthcare research in low-resource contexts (8).

For example, Radin et al. (5) used wearable data to forecast rates of influenza-like illness. Using de-identified Fitbit data on heart rate, sleep, and weekly estimates of influenza-like illness (ILI) rates at the state level (reported by a US authority), they could significantly improve predictions. Authors thus emphasized the potential of wearable data for fast outbreak response. Based on this work, the Robert Koch Institute, the German research institute for disease control and prevention, launched the so-called “Datenspende” study (6). With de-identified wearable data donated by German citizens (“Datenspende”), they were able to predict regional probabilities of COVID-19 outbreaks. Incorporated were data on pulse, physical activity (PA), and sleep, as well as weather data. The development of predictive models from wearable data, such as body shell temperature, could aid in the control of the rising burden of communicable and non-communicable diseases in high-exposure countries, such as sub-Saharan Africa (5). Wearable devices might also effectively identify patients at risk and enable better patient monitoring and health care in rural settings (8).

Heart rate (measured with standard, non-wearable devices) has previously been able to predict all-cause mortality of vulnerable populations in sub-Saharan Africa (9, 10). These punctual/point measurements were only conducted in clinic. Wearable devices might not only generate long-term data automatically but are low cost generated in the natural environment of patients (2). They might also effectively identify patients at risk and enable better patient monitoring and health care in rural settings (8). Overall, insights into activity, morbidity, and vital patterns could be starting points for tailoring and targeting of public health and behavior change interventions (11, 12). A few projects are already underway in this regard; like the International Physical Activity and the Environment Network (IPEN) project (13), which may be the largest study to date using wearable devices to track movement in different countries and continents, has been used to design activity-friendly built environments (14).

Despite the fact that the potential and usefulness of wearables in rural and low-resource contexts have been widely identified (1, 2, 8), most research has been undertaken in high-income contexts (1). Wearable devices are used in a variety of ways in high-income research contexts, including testing novel technologies, producing population-based insights, employing wearables in treatments and monitoring, etc. (1). There are limited insights on individual and cultural acceptability and (technical) feasibility of wearables in LMICs settings. Few studies have been conducted to date, most of them mainly qualitative (not including wearable device data) or using high-end, non-consumer-based wearables. For example, Larnyo et al. conducted a qualitative study on the general preparedness of Ghanaian family caregivers of dementia patients to employ wearables. They found that caregivers were willing to recommend the usage of healthcare wearable devices for dementia patients (15). Davies et al. evaluated a wearable device and home-based sensor for monitoring epilepsy in children in South Africa and found their proof-of-concept study provided beneficial outcomes for remote patient monitoring (15, 16). However, they reported issues with wearable management and internet connectivity in the field (16). Wearable photoplethysmography measurements may be impeded by variations in ambient conditions (e.g., heat exposure), everyday activities (e.g., farming), and signal crossover (17–20). More research is needed to understand the effect of skin pigmentation and its interference with measurements; some studies found no evidence (17, 20), while others did (21). Furthermore, wearables must be accepted or even desired by users in order to generate meaningful measurements (22). Thus, insights into the feasibility of wider applications of wearables, acceptability, and technological reliability in rural settings are still limited.

### HDSS is ideal for population health surveillance and as a launching point for introducing wearables as a routine measurement to capture individual-level health effects

In rural, low-income regions, health and demographic surveillance systems (HDSSs) are an ideal starting point for evaluating wearable devices. Each HDSS represents a dynamic population cohort that varies over time frequently based on entry (birth, in-migration) and exit (death, out-migration) events. Through routine data collection, longitudinal databases of individuals and social units are collected in areas where vital events registration and health information systems are weak or non-existent. The HDSS provides a well-structured platform to collect valid and reliable population-based data, particularly in areas where vital events registration and health information systems are weak or non-existent. In rural, low-resource contexts, HDSS provides an infrastructure for conducting individual studies (23, 24) and implementing consumer-based wearable devices for population research.

This case study seeks to understand if consumer-based wearables could be used to generate longitudinal, high-quality individual-level health data in vulnerable populations in LMICs using two wearables: (i) Withings Pulse HR fitness tracker wristband (WPHR) and (ii) the Tucky axillary thermometer patch (thermometer patch). Specifically, we aimed to (i) evaluate the feasibility of using wearables in rural communities in the Nouna HDSS in Burkina Faso (25) with regard to data quality (plausibility of output values) and quantity (data completeness) and (ii) understand acceptance among the study population.

Our specific objectives were to:

(i) understand study participant and field worker acceptability of wearable devices with respect to hindering and enabling factors, and
(ii) evaluate data quantity (i.e., data completeness and its potentially influencing factors) and quality (i.e., the plausibility of output values) of wearables within the context of the Nouna HDSS to understand individual sleep, activity, and heart rate characteristics within vulnerable populations.

## Methods

This case study used a mixed-methods approach with a convergent explanatory design (26, 27) in which qualitative data complemented quantitative data. Our results are reported in line with the Consolidated Standards of Reporting Trials (CONSORT) extension for randomized pilot and feasibility trials (28) (Supplementary material S1). This study is further detailed in the protocol paper; for details, see (4).

### Study location: The Nouna HDSS in Burkina Faso

As part of the INDEPTH network, the HDSS in Nouna, Burkina Faso, managed by the Centre de Recherche en Santé de Nouna (CRSN), gives access to retrospective health and population data encompassing about 115,000 individuals over 20 years (25, 29). Since 1992, the Nouna HDSS has been managed by the CRSN, a Ministry of Health-affiliated research institute. Burkina Faso is located in sub-Saharan Africa and has one of the highest burdens of climate-sensitive diseases. The surveillance area of the Nouna HDSS is characterized by a tropical climate with one rainy season lasting from May to September (mean annual rainfall of 800 mm) and year-round high temperatures. Malnutrition and malaria are common in the Nouna HDSS (25).

### Sampling and study population

We calculated a sample of *n* = 150 participants, based on a rounded population size of *n* = 100,000 (total HDSS population excluding children under the age of 6 years), a confidence level of 95%, and a margin of error of 8%. This sample size was deemed adequate and consistent with the available literature (30) for estimating acceptability in this population and evaluating feasibility. Eligible were all HDSS inhabitants older than 6 years, willing to participate and wear the wearables. Participants were randomly assigned to two study arms.

Seven villages within a closer range (walking distance below 30 min) to a health facility were randomly selected from the HDSS database. Field workers were assigned villages in close proximity to each other to optimize data collection. We conducted purposive block randomization with the existing HDSS population and randomly drew *n* = 170 individuals through the database (n = 150 study population, oversampling of *n* = 20). Refer to the protocol paper for exact details on randomization and sampling (4).

### Study procedures

The case study was conducted at the Nouna HDSS from January 2021 to March 2021, enclosing three study cycles with each n = 50 study participants (1st cycle: 18/01/2021–07/02/2021; 2nd cycle: 08/02/2021–28/02/2021, and 3rd cycle 1/03/2021–21/03/2021).

In each study cycle, *n* = 27 out of 50 participants (study arm 1) were instructed to wear the WPHR all day, while *n* = 23 study participants (study arm 2) also wore the thermometer patch at night (to determine if wearing multiple devices affects acceptability and data completeness). Every 4 days, study participants met with a field worker to complete an acceptability questionnaire, synchronize data, and charge the WPHR battery (study arm 1). Study participants who wore a thermometer patch received a smartphone and a portable solar panel to charge their devices during the study period. Participants' vital parameters were remotely monitored *via* the wearable platforms, so participants with abnormal measurements could be referred to a health facility.

The project was designed in close collaboration with the CRSN team and community leaders, like village chiefs and household heads, to ensure their awareness and acceptance. The field team contacted study participants, as well as family members and community leaders, to inform them about the study and obtain informed consent *via* fingerprint [for details, see protocol paper (4)]. Study participants could withdraw their participation at any time. Consistent with other HDSS-related studies, participants received the US $6 for their participation.

#### Wearables

Table 1 outlines the details of the two employed wearables. Reasons for selecting these devices include the low cost to facilitate population health surveillance, user-friendliness, functionality, and validity [for details, see (4)].

**Table 1:** Details on Withings Pulse HR fitness tracker (WPHR) and the Tucky thermometer patch [adopted from the protocol (4)].

	**Withings pulse HR**	**Tucky thermometer**
Consumer-grade wearables part of the feasibility study	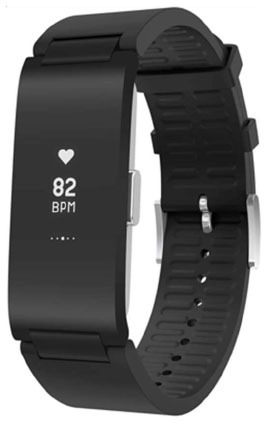	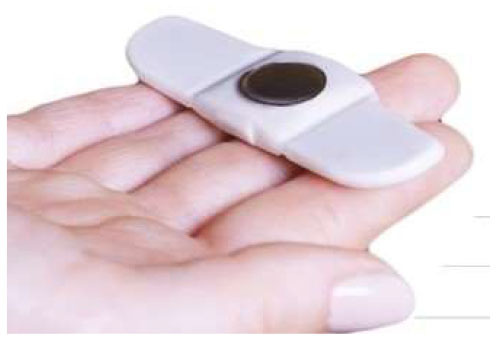
Measures	**- Steps (distance and kilocalories):** • Measured continuously with accelerometery (impact of the foot on the ground, exact algorithm undisclosed) - **Activity:** • Activities like walking, running, swimming, cycling, and different sports (soccer, fitness, boxing, basketball, squash, etc.) are detected with accelerometery, algorithm undisclosed **- Heart rate:** • Routinely measured every 10 min with photoplethysmography (exact algorithm undisclosed) • Measurement every 1 s if workout detected **- Sleep** • Based on accelerometer (i.e., sleep is calculated based on the absence or decrease of movements, exact algorithm, and thresholds for awake/sleep undisclosed; the sleep quality algorithm considers the following factors: sleep duration, sleep depth (calculated with movement intensity), regularity (uniformity of bedtime and rising time), interruptions (waking phases as identified by the wearable)	- **Body temperature (shell temperature)** • Measured continuously with contact sensor
Wear location	Wrist	Under right armpit
Wear frequency	During the whole study cycle	During night
Data synchronization	5 days of local data storage between synchronizations (within 10 m of tablet)	Requires regular synchronization (within 10 m of tablet)
Connectivity	Bluetooth low energy	Bluetooth low energy

While the participant conducted the questionnaire with the field worker, the wearables automatically synchronized with the tablet of the fieldworker which then uploaded the data to the respective wearable platforms. Data obtained from the Withings platform (31) included heart rate, physical activity [e.g., measures of steps, distance, calories burnt, activity categories (automatically classified by the WPHR)], and sleep measures (duration, awakenings, and sleep quality; WPHR output, exact preprocessing undisclosed). Body shell temperature data were downloaded from the Tucky platform (32). For both wearables, the specifics of data preprocessing were unavailable and we had no access to raw data. Furthermore, we collected data on sex, date of birth, weight, height, and blood pressure.

#### Questionnaire

We developed a 5-item Likert-scale questionnaire with multiple-choice (MC) and open-ended questions regarding: (a) participant demographics, (b) ease of use of wearable as reported by field worker, (c) study participants' acceptance of wearables, and (d) daily self-reported activity of study participants (Supplementary material S2). The questionnaire is based on established, applied questionnaires such as (33) and (34) and was adapted to the study setting. The survey was conducted using Survey Solutions (35), a freely available survey software that was run on our local project server. The field staff asked questions to the participants who collected responses on their tablets (except for three multiple-choice (MC) and one open-ended question answered by the field staff). Therefore, unless otherwise stated, questionnaire results refer to participants responses. Fieldworkers visited participants every 4 days at times and locations that were convenient for each participant; thus, each participant completed five questionnaires during the course of one study cycle. Furthermore, after receiving consent, the first author (SH) conducted informal feedback meetings with study managers.

### Data analysis

#### Objective 1: Acceptability—Hindering and enabling factors

We analyzed qualitative data (open-ended questions and informal feedback sessions with stakeholders and field workers) convergent with quantitative data (Likert-scaled and MC questionnaire responses, wearable data), to facilitate a better understanding of quantitative data and findings. Responses to Likert-scale and MC question items were analyzed descriptively, while open-ended questions were coded thematically (36–38). We followed the steps “compiling, disassembling, reassembling, interpreting, and concluding” (39). Data were cleaned in Excel. After repeatedly reading responses (familiarization), themes were inductively identified from questionnaire responses.

#### Objective 2: Quantity and quality of wearable data to understand individual sleep, activity, and heart rate characteristics within vulnerable populations

R was used for analysis and visualizations (40). Demographic, wearable, and survey data were descriptively summarized as mean (standard deviation) or median (first quartile, third quartile). Categorical variables were counted and provided as numbers (percentage). We used quantiles to split age groups (i.e., minimum, 25th, 50th, 75th, maximum).

We refer to data coverage as the proportion of the study for which wearable data were collected (with an acceptable data rate for the respective data output rate). Literature (41–44) and sampling rates of wearables used in our study (Table 1) guided our analysis, so a single data point covered the following epochs (i.e., the following time spans were tolerated between two measurements and seen as time covered by data):

- accelerometer data (activity): 60 min [During the continuous activity, output data are sampled every second; however, the wearable does not distinguish between inactivity and non-wear, both of which result in data output gaps. As similar issues exist with research-grade accelerometers, we use the commonly utilized interval of 60 min (41, 42) as maximal inactive time without measured movement still designated as sedentary/inactive time; everything over 60 min without movement is therefore regarded as time not covered by data, i.e., missing data. Thus, a single output value may cover 60 min; everything above 60 min between two values is considered missing for time, or missing data.].
- body shell temperature: 5 min (output data frequency: every minute when attached for sleep, the rest of the time is tolerance, i.e., anything more than 5 min between two output data values is considered missing data).
- heart rate: 15 min (output data frequency: every 10 min, rest is tolerance; i.e., anything more than 15 min between two output data values is regarded as missing data).

For data coverage of each participant and each day, we calculated the difference between two measurements, deducted the tolerances (i.e., 60/5/15 min for accelerometer/body shell temperature/heart rate data; see above), and added all values >0 (i.e., the excess of study time, not covered by a data point and the tolerance interval).

The thermometer patch was only worn during sleep, and its duration of use varied among study participants; a daily average of 8 h of sleep was used to calculate data coverage (45). Thus, the calculated time not covered by data was subtracted not from the total duration of the study (24 h × 3 weeks) but from a duration that had been adjusted for data coverage (8 h × 3 weeks). We did not use the WPHR sleep data as a reference because the data completeness of the WPHR is being investigated in this study, and the data may be inaccurate and inconsistent across individuals (e.g., one might only want to wear one wearable). Despite the fact that the 8-h reference may be inaccurate due to individual sleep variations, the data coverage of the thermometer patches was also consistent when using references for sleep duration other than 8 h (Supplementary material S3A).

## Results

**Figure 1:**
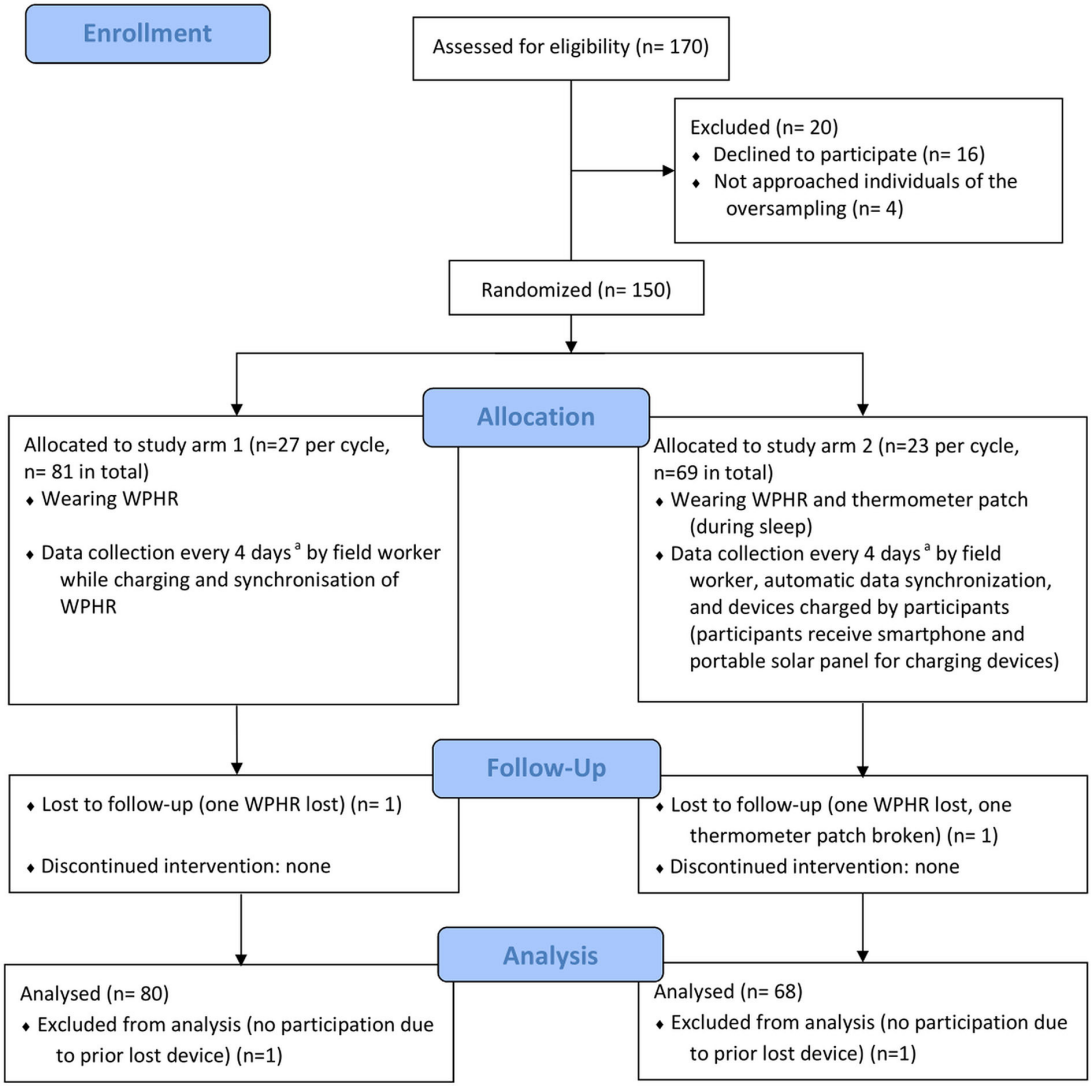
CONSORT flow diagram (28) (^a^depending on weekends and public holidays).

**Figure 2:**
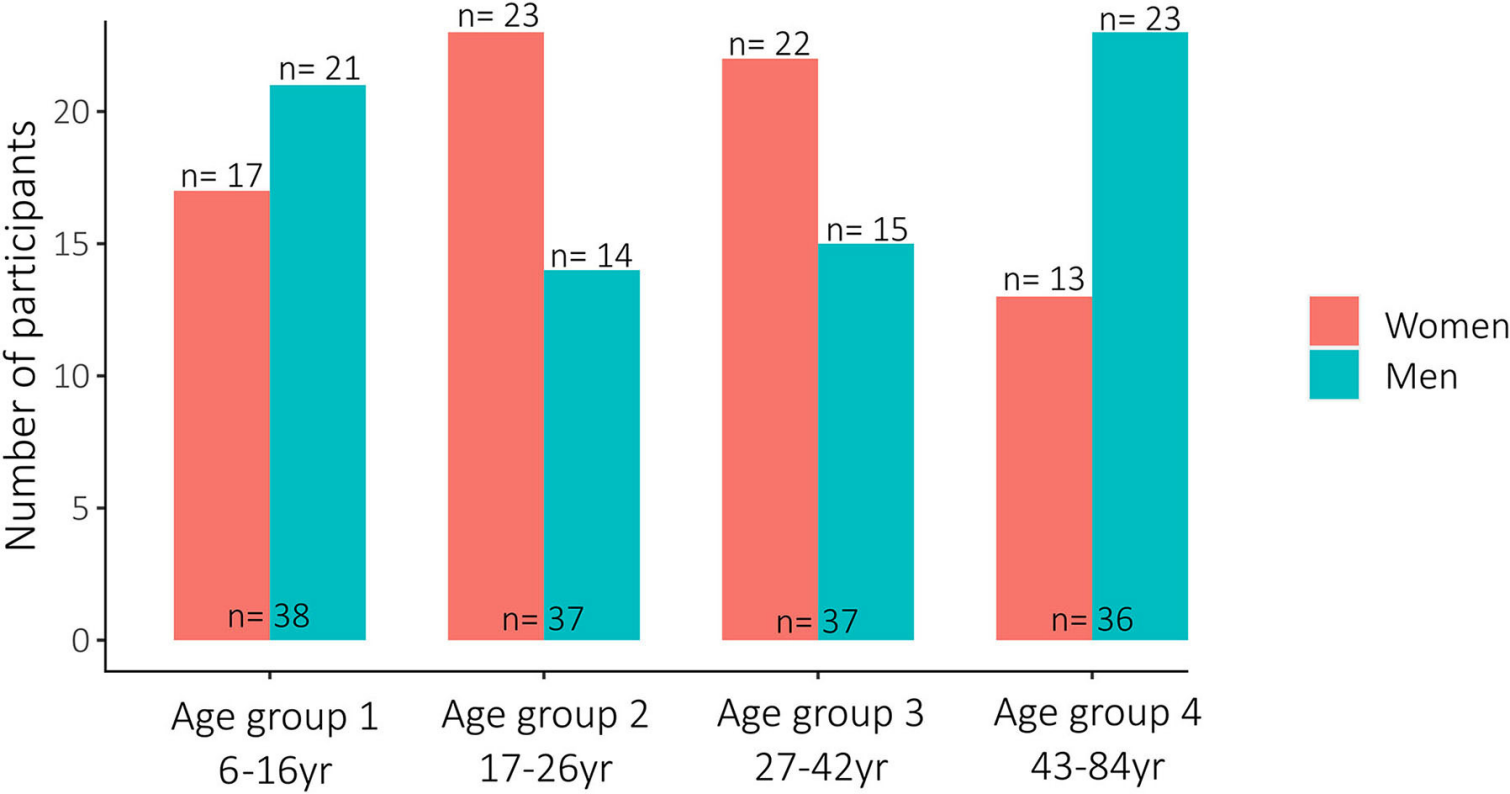
Overview of all study participants per age groups and sex.

The study involved *n* = 148 participants (see Figure 1). A total of *n* = 73 (49%) women and *n* = 75 (51%) men were included (see Figure 2), with a median age of 26 years (range 6–84 years); *n* = 80 (55%) only wore the WPHR, and *n* = 67 (45%) additionally wore the thermometer patch. A total of *n* = 16 individuals (women *n* = 9, men *n* = 7) refused to consent; oversampled study participants were approached (see Figure 1). In the second study cycle, two WPHRs (4%) were lost and one thermometer patch (4%) was damaged. Therefore, the third cycle comprised *n* = 48 study participants, i.e., one participant less per study arm (see Figure 1). Three further patches (13%) were damaged at the end of the last cycle.

### Objective 1: Acceptability—Hindering and enabling factors

A total of 841 questionnaires containing responses from study participants and field workers were collected. Likert-type items were grouped into three categories: (i) acceptance toward wearable, (ii) acceptance toward wearing two wearables, and (iii) technical feasibility (answered by field workers).

**Figure 3:**
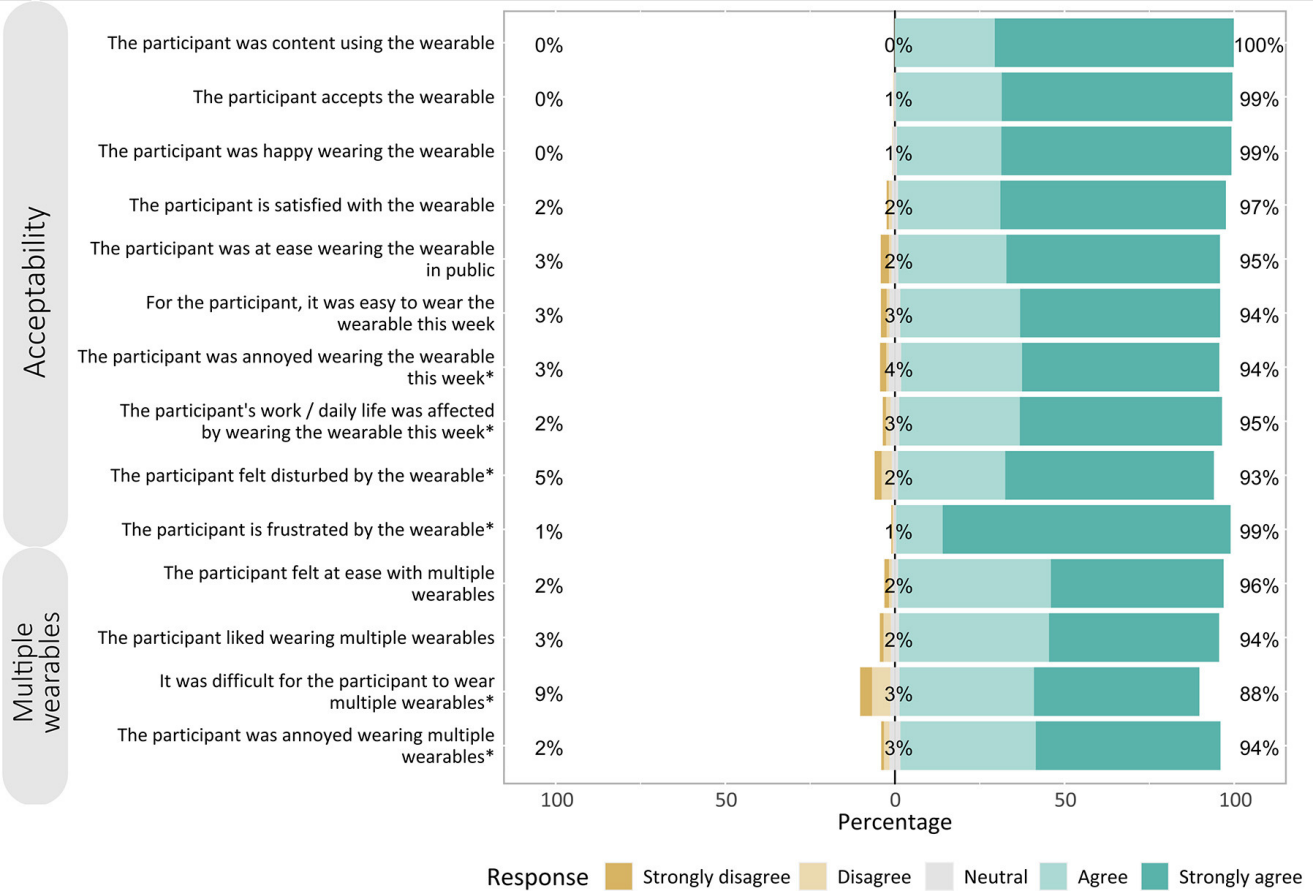
Responses to Likert-type questions. Likert-type responses and their percentages according to the three categories: (1) acceptability of wearing a wearable, (2) wearing multiple wearables at once, and (3) technical feasibility of implementation and maintenance. Items with negative statements (marked with *) have reversed scales for readability and comparability.

Fourteen out of 17 question items had 90% or higher positive agreement (“Agree” and “Strongly agree” responses) (see Figure 3). The majority of study participants agreed on acceptability, with agreement ranging from 94 to 100% (mean: 97%).

Agreement on questionnaire items was comparable across subgroups of sex, age, study arms, and study cycles (Supplementary material S3B).

#### Field worker's feedback

Field workers reported informally that they frequently observed participants enjoying the wearables, e.g., considering them as “cool.” On the other hand, field workers reported concrete technical issues such as internet connection issues, data synchronization issues between wearable and tablet, and the patch falling off. Two of nine field workers (22%) minimally once responded “Disagree” or “Strongly disagree” when asked if using a tablet to manage data was difficult. Nevertheless, for many field workers, handling data was straightforward (synchronization) (*n* = 8, 89%), as was using the tablet-based wearable application (*n* = 9, 100%).

#### Quality-impeding questionnaire items

MC and open-ended questions led to varied responses (see Tables 2–**4**). The majority of study participants (*n* = 122, 83%) were not irritated by wearable devices, and they were often overlooked (*n* = 51, 35%). Ten participants (*n* = 10) found the thermometer patch difficult to wear, and seven (5%) needed extra time to handle the wearables. A few participants (*n* = 3, 2%) felt limited in their movement by the wearables or removed them (*n* = 1, 1%); one (1%) experienced increased transpiration. One female participant (1%) of age group 3 (27–42 years) had palpitations while wearing the WPHR (she was referred to a health facility without diagnosis).

**Table 2:** Overview of key responses to multiple-choice and open-ended questions about participants' general experiences with wearables and effect on sleep.

	**Answers**	***n***	***n*%**	**Quotes**
Experiences	**What did you experience and feel when wearing the wearable?**			
	I was not disturbed	122	83%	“It's like a watch [Withings], not disturbing”
	I forgot that I wear it	51	35%	“Wearing the wearable had no effect on me,” “I forgot it”
	Sometimes difficult	10	7%	“The Tucky is difficult to wear”
	It needed time/attention	7	5%	“I paid extra attention not to damage it”
	The wearable limited my movements	3	2%	
	Removing wearable	1	1%	“I just wanted to remove it,” “I had to take it off for some days because it was itchy”
	Transpiration	1	1%	“It adhered to my skin [Withings]”
	Other	1	1%	“I had heart problems [palpitations while wearing the Withings]”
Sleep	**Did wearing the wearables have an effect on your sleep?**			
	Yes	7	5%	
	No	136	93%	
	**What effect occurred because of the wearable device during your sleep?**			
	The wearable fell off during the night	4	3%	“The adhesive of my Tucky did not adhere well”
	I woke up sometimes	4	3%	
	I woke up often	2	1%	“I woke up often to drink to calm my palpitations,” “The Tucky disturbs my sleep”
	I could not sleep at all	1	1%	“I [felt like I] could not turn”

Analyzed were n = 841 valid questionnaires of n = 148 participants.

Most participants (*n* = 136, 93%) reported that the wearable had no effect on their sleep. Some (*n* = 4, 3%) noted that the thermometer patch came off during the night. Others (*n* = 4, 3%) woke up during the night, while one (1%) had trouble sleeping.

The study participants perceived the look and wear of the wearables as positive (see Table 3), including weight (*n* = 89, 60%), wear comfort (*n* = 83, 56%), appealing device appearance (*n* = 76, 51%), size (*n* = 73, 49%), or practical wear (*n* = 52, 41%). Study participants (*n* = 16, 19%) also enjoyed the wearable in general and the information it provided (*n* = 2, 1%). Some (*n* = 7, 5%) reported difficulties wearing the device, particularly the thermometer patch not adhering well to the skin, as well as the wearables being too big (*n* = 1, 1%) or being frequently asked about it by friends (*n* = 1, 1%).

**Table 3:** Overview of key responses to multiple-choice and open-ended questions about perceptions on wearables (of participants and social circle).

	**Answers**	***n***	***n*%**	**Quotes**
Participant's perceptions	**What did you like about the wearables?**			
	Good weight	89	60%	
	Easy/comfortable wearing	83	56%	“I like the wear of the wearable”
	Looks nice	76	51%	“That is a nice watch”
	Good seize	73	49%	“I like the shape”
	Practical/handy to wear	52	35%	“It's just like a watch you can wear everyday”
	Just liking the wearable	30	19%	“I enjoy the wearable,” “It's amusing,” “I like the wearable”
	Informative	2	1%	“The wearable enables me to see and follow my daily activity and energy expenditure,” “It helps me control my health”
	**What did you not like?**			
	Difficult to wear	7	5%	“The Tucky is difficult to wear [and keeping adhered]”
	Too big	1	1%	
	Other	1	1%	“My friends disturbed me a lot as they also wanted to wear and see the it [Withings]”
Peer perceptions	**Did people ask you about the wearable?**			
	Yes	41	25%	
	No	107	73%	
	**About what wearable did the people ask?**			
	Withings	38	25%	
	Tucky	10	6%	
	**What questions did they ask?**			
	Aim of wear, functionality	25	17%	“Why do you wear that,” “That is a nice watch, […] what is it for?,” “What is the aim of wearing this watch?,” “What is it doing?,” “Is it a toy?,” “Its purpose [?]”
	Medical/health beliefs	19	13%	“Is it because of HIV that they gave you this,” “People asked if that is how they control COVID with,” “If it's a medicament,” “Is it for [your] health?”
	What is it?/curiosity and desire to touch	14	9%	“What is it, … let me try,” “They were curious and wanted to touch,” “What kind of watch is it?,” “They asked what I am wearing”
	Where from?/acquiring	11	7%	“Do you sell it? Do you have it in stock?,” “How can someone acquire such a watch?,” “Did you buy it?,” “You have a nice watch, who gave it to you?,” “Is it from the market?”
	Side effects on health	5	3%	“If it's a magical watch?,” “Is it dangerous to wear?,” “They asked if it makes me ill,” “If it has side effects”
	People recognizing the study participation through the wearable	3	2%	“You also got ‘their’ watch?,” “Children also wear that [study] watch? Who gave it to you?,” “So you also have one of these watches, no?”
	Details on wearing	3	2%	“They asked if it's disturbing,” “[Some asked] if I constantly wear it even while taking a shower?,” “If I feel at ease when wearing the wearable”

Analyzed were n = 841 valid questionnaires.

The peer perceptions of wearables varied. Family and friends of some study participants (*n* = 41, 25%) were curious and asked about the wearables. Common questions were about the device's purpose and why they wear it (*n* =1 9, 13%), as well as possible medical applications like monitoring or healing COVID-19 (*n* = 14, 9%). Few people enquired about details about wearing the wearable and wanted to touch it and try it on (*n* = 14, 9%). People also asked what the wearable is (*n* = 11, 7%), how it was acquired (*n* = 5, 3%), and about any side effects, also in relation to magic (superstition) (*n* = 3, 2%). Some recognized the wearable from another study participant they had met and were curious about the study and how they could participate (*n* = 3, 2%).

In 95% of the cases (*n* = 140), study participants reported no challenges with the wearable (see Table 4). Challenges were mostly of technical nature such as broken devices or synchronization problems (*n* = 5, 3%), as well as perceived limitation of movement (*n* = 1, 1%). Two participants (1%) experienced itching.

**Table 4:** Overview of key responses to multiple-choice and open-ended questions about experienced challenges with the wearables, and hindering factors for a possible long-term study with wearables.

	**Answers**	***n***	***n*%**	**Quotes**
Challenges	**Did you experience challenges with the wearable/s this week?**			
	Yes	4	3%	
	No	140	95%	
	**What challenges did you experience with the wearable/s?**			
	Technical	5	3%	“[My] Tucky is not working anymore,” “The display of the Withings does not light up anymore,” “Problems with synchronization, we had to take the Tucky to Nouna to synchronize it with another device”
	Limiting movements	1	1%	“Sometimes hindering”
	Pain caused by wearable	1	1%	“I had heart problems [palpitations]”
	**Did you remove the wearable/s this week?**			
	Yes	7	3%	
	No	141	96%	
	**Why did you remove it?**			
	Device broke/technical issue	5	3%	“The bracelet of the Withings broke,” “The Tucky does not work anymore,” “When the Tucky fell off, I did not wear it anymore,” “The participant just reset the tablet “[field worker],” “We had to take the device to Nouna because it was not synching [field worker]”
	Only temporary removed due to daily life/routine	4	3%	“I removed it when having morning sickness [due to pregnancy],” “I removed the wearable for taking a shower because I feared to spoil it,” “For charging”
	Itching	2	1%	“I had to remove it due to itching”
Hindering factors for	**What would be obstacles for you to wear the wearable for a longer study period (i.e., a year)?**			
long-term wear	No problem at all	53	35%	“I am available no matter the study duration!!,” “I am always available if you need me [for the study],” “I am absolutely okay with a longer period as I feel at ease wearing the wearable,” “There are no obstacles as I feel at ease wearing the wearable”
	Study period was too long	48	32%	“[I] can't wear it for a very long time,” “Impossible,” “Yes I would have a hard time wearing for such a long period”
	Participation/questionnaires consume too much time	46	31%	
	Familial, social acceptance	13	9%	“My husband would be the problem,” “If I have the permission of my husband,” “If my father gives his permission,” “It depends on the decision of my parents,” “If my husband gives his permission again”
	Affecting daily life and activity (negatively)	5	4%	“When washing, I paid extra attention not to spoil it,” “It hinders me doing everything”
	Not informative enough concerning health	4	3%	“I am discouraged by the fact that the Tucky does not give any information [on a screen]”
	Possible side effects	4	3%	“[I] fear side-effects or long-term consequences”

Analyzed were n = 841 valid questionnaires.

Almost all study participants (*n* = 141, 96%) reported wearing the wearables continuously during the current study week. When asked if they could imagine wearing the wearable for a longer period (i.e., a year), a third (*n* = 53, 35% 281/841) answered there would be no problem at all, while a third (*n* = 48, 32%) said that 1 year would be too long, and the participation, i.e., the weekly questionnaires, would take too much time (*n* = 46, 31%). Furthermore, some (*n* = 13, 9%) perceived familial and social acceptance as uncertain. Negative effects for daily life (*n* = 5, 4%) included paying greater attention when wearing the wearable for fear of damaging it. Some participants (*n* = 4) desired more health information, i.e., a screen on the thermometer patch. Others feared adverse effects (*n* = 4, 3%).

### Objective 2: Quantity and quality of wearable data to understand individual sleep, activity, and heart rate characteristics within vulnerable populations

Regarding the results of the questionnaires and the feedback from field workers, data missingness was largely attributable to two factors: (i) incorrect or non-wearing of the wearable by study participants and (ii) technical difficulties like synchronization issues and measurement failures.

We found a wide range of missingness, ranging from 0 to 100 % data coverage. Accelerometer data were most complete, with higher missingness for heart rate and body shell temperature data (see Table 5, and for more details, see Supplementary material S3C). Mean data completeness for accelerometry was 43%, heart rate 3%, and body shell temperature data 4%. Among all 148 participants, *n* = 20 participants (14%) had <1% data completeness for accelerometry and *n* = 96 (65%) for heart rate data. Of *n* = 68 participants (68/148, 46%) who wore the thermometer patch, *n* = 51 participants (75%) had <1% data completeness (Table 5).

**Table 5:** Data completeness of the variables: accelerometry, heart rate, and body shell temperature for all study participants for the complete 9-week study period.

	**Mean data completeness**	**Max data completeness**	***n* (%) participants with data completeness <1%**
Accelerometry data	43%	100%	20 (34%)
Heart rate data	3%	43%	96 (65%)
Tucky temperature data	4%	59%	51 (75%)

**Figure 4:**
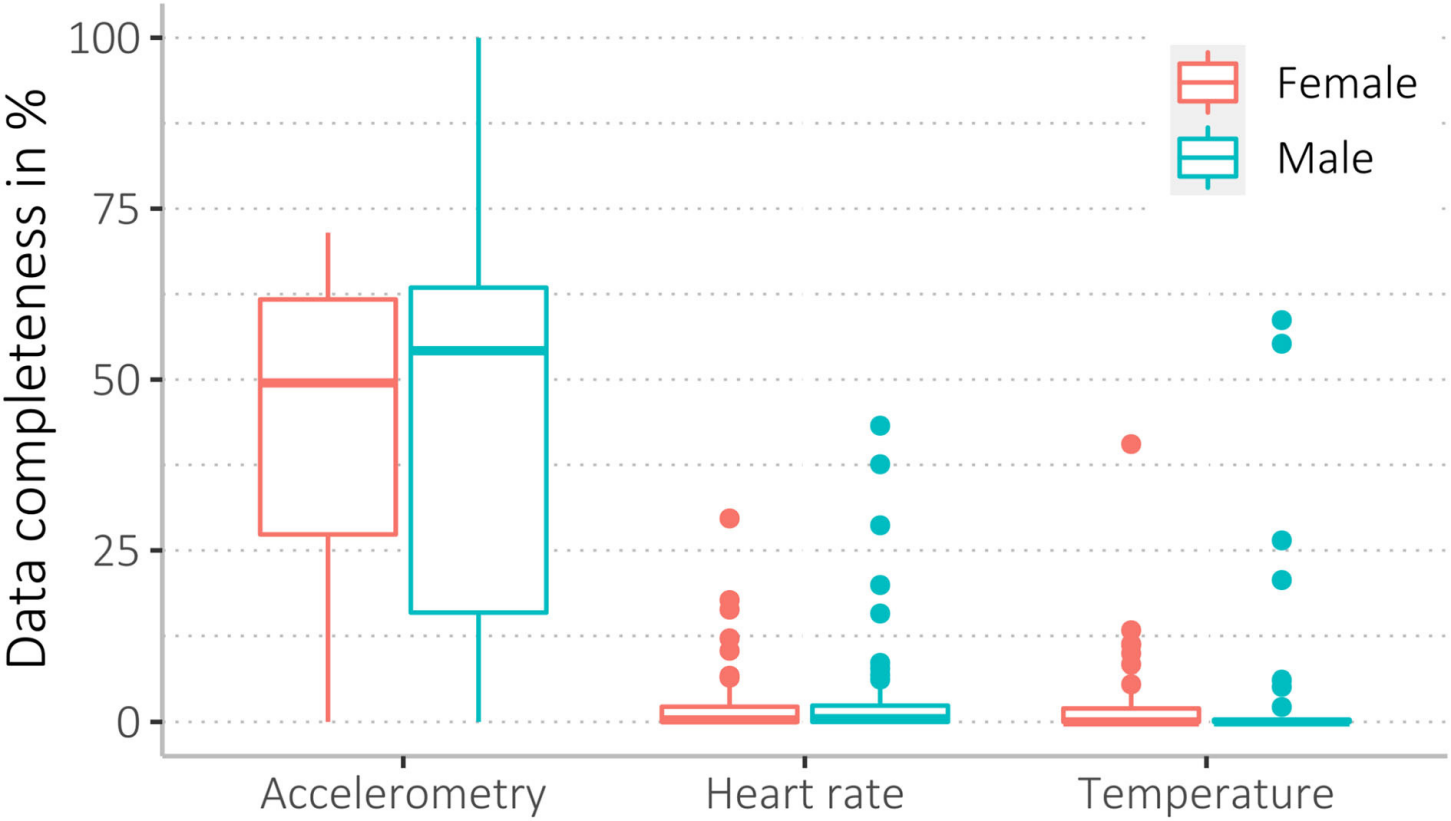
Data completeness of female and male participants regarding the three data sources (accelerometry, heart rate, and temperature).

**Figure 5:**
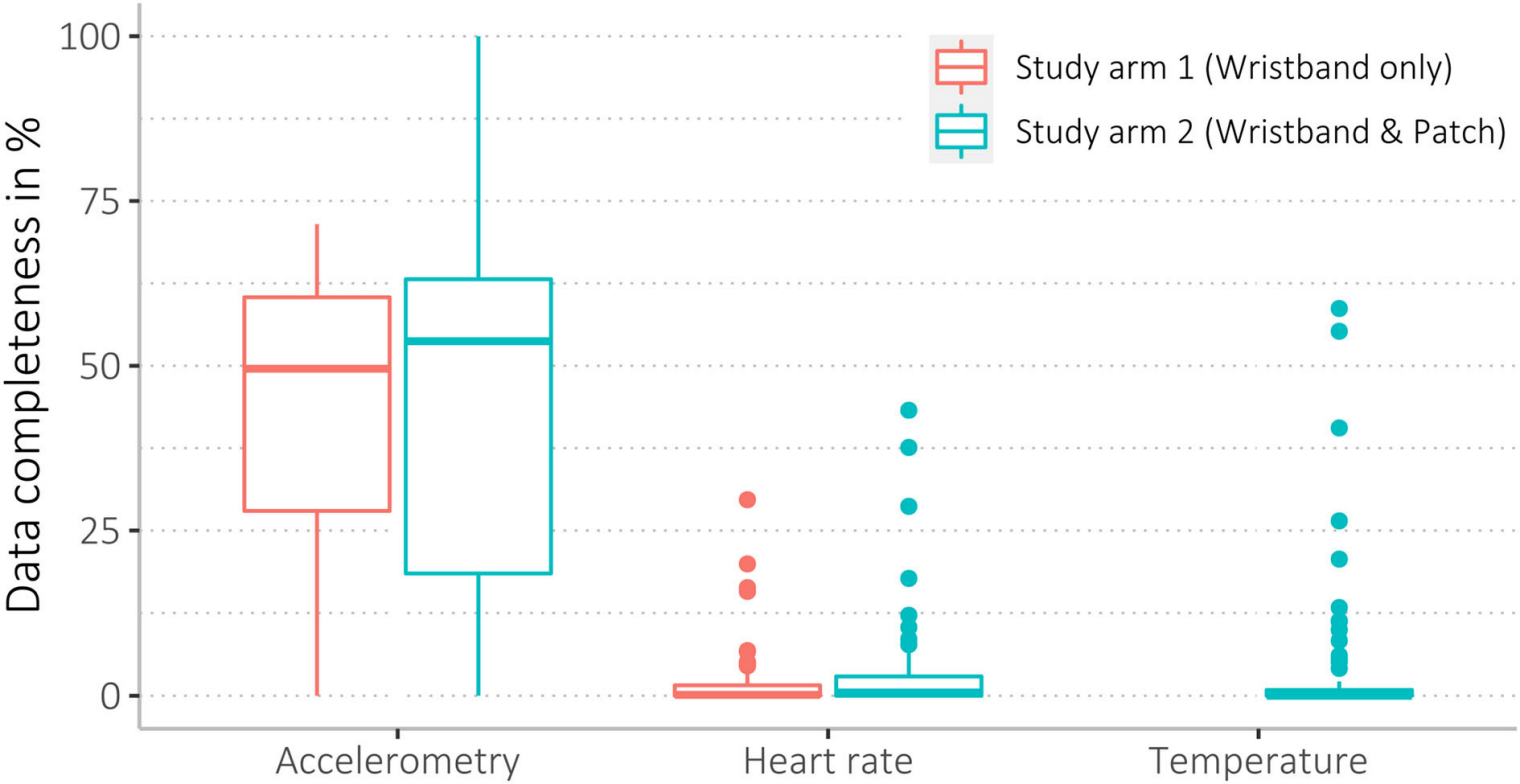
Data completeness of study arms. As the participants from study arm 1 only wore the WPHR, there is no temperature data for this group.

**Figure 6:**
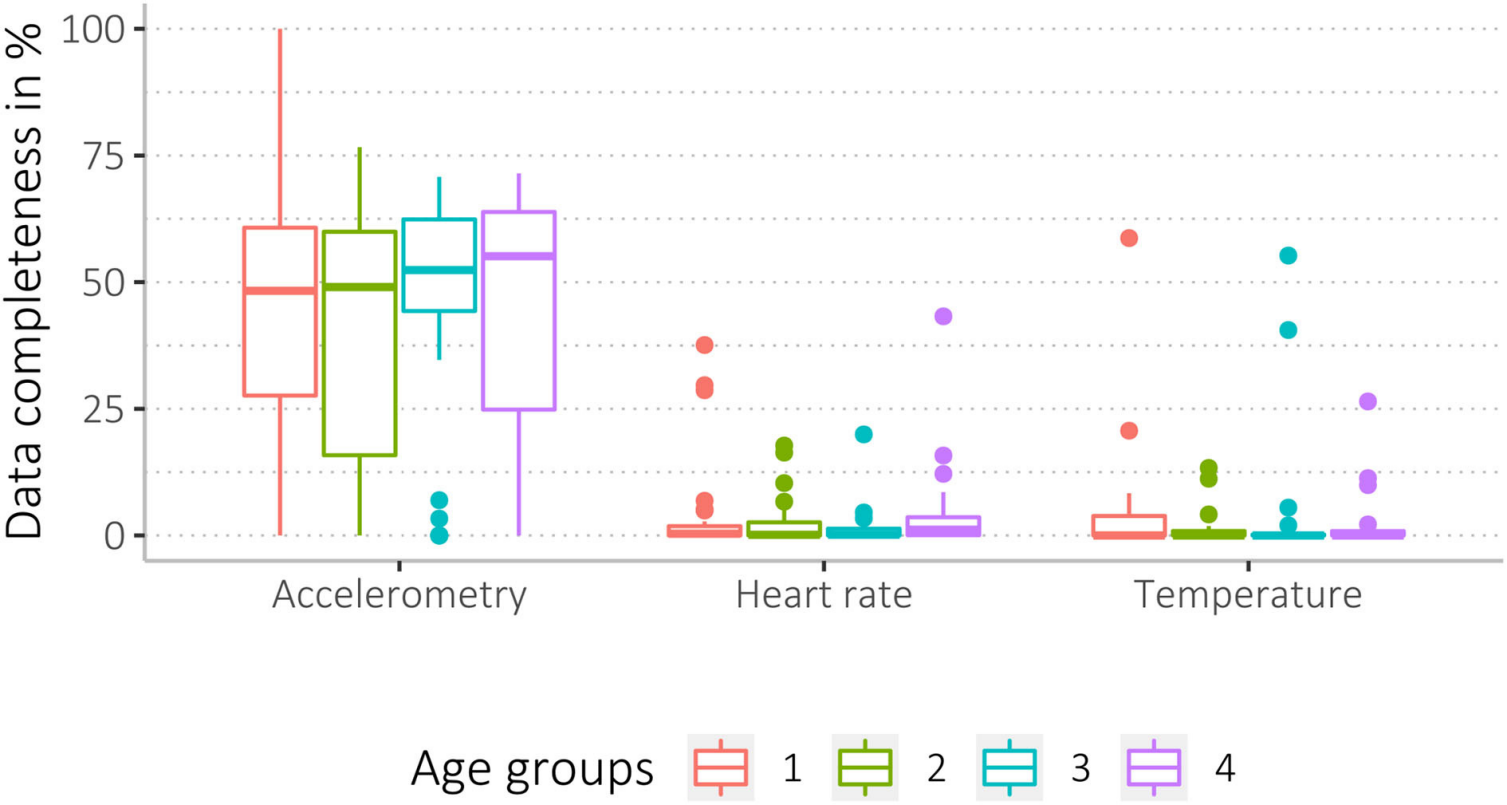
Data completeness of the four age groups (6–16 yrs, 17–26 yrs, 27–42 yrs, and 43–84 yrs) regarding the three data sources (accelerometry, HR, and temperature).

Data completeness between sex and age groups, as well as study arms and acceptability levels, was similar (see Figures 4–6, Supplementary material S3C).

**Figure 7:**
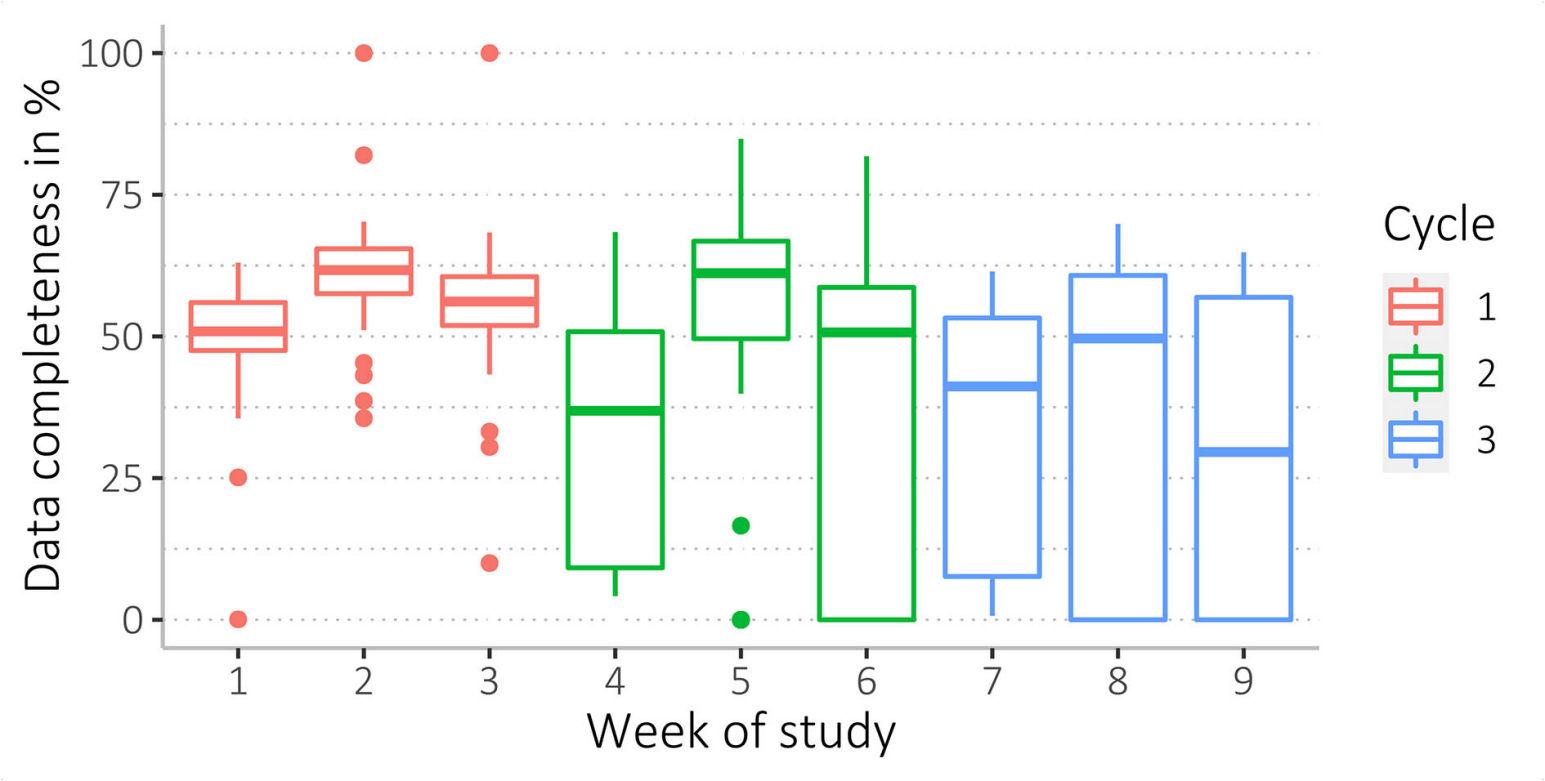
Completeness of accelerometry data collected during the complete study (duration of 9 weeks).

**Figure 8:**
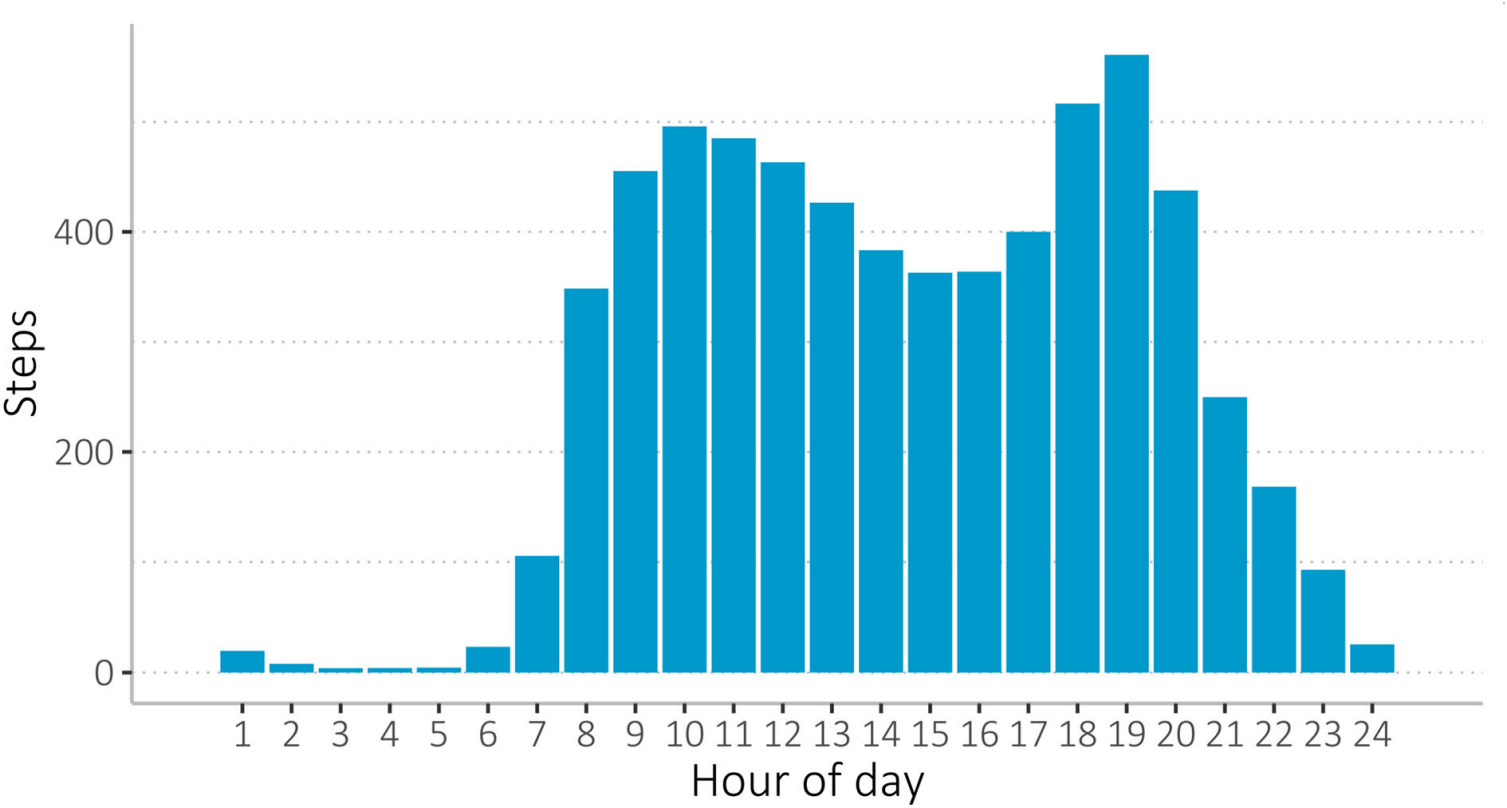
Overview of average steps taken during the day per study participant (average of all study participants over the full 9-week study period), measured with the WPHR wearable. During the hottest part of the day, there is a drop in activity between 12 pm and 6 pm.

The mean data completeness decreased from 53% in the first cycle to 31% in the last cycle regarding accelerometer data (see Figure 7 and Supplementary material S3C). The second week in cycles had the highest data completeness (Figure 8).

#### Wearable data quality: Individual sleep, activity, and heart rate

**Figure 9:**
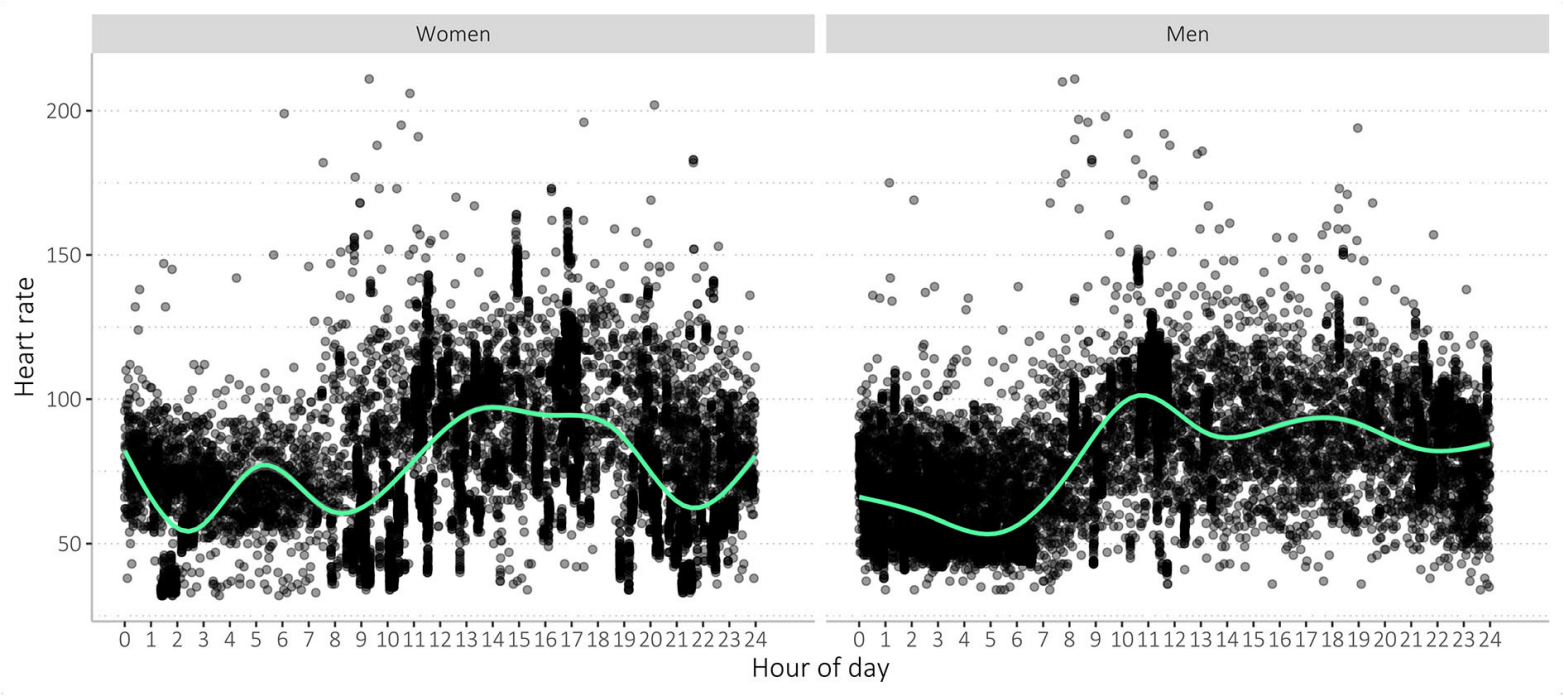
Overview of participant's heart rate measurements. As measured by the WPHR wearable device, all study participants over the full 9-week study period were displayed per gender. Measurements were widely variable, and the values were lower during the night.

Overall, activity, heart rate, sleep, and body shell temperature values were widely spread (see Table 6, Supplementary Figures 9, 10, Supplementary material S3D). On daily average, study participants burnt about 1,300 kilocalories (kcal), walked over 8,000 steps, covered a distance of 5, 6 km and slept 7 h (Table 6). Heart rate averaged 73 beats per minute (bpm). On average, the participants' activity levels decreased in the afternoon (Figure 9). Participants woke around 7:00 and went to sleep between 22:00 and 23:00 (Supplementary material S3D). Most body shell temperatures ranged between 34 and 37°C, with some outliers (Supplementary material S3D).

**Table 6:** Summary of wearable measurements, including heart rate, energy expenditure, steps, and distance (in meter) covered per day measured with the fitness tracker and body shell temperature (in °C) measured with the thermometer patch.

	**Min**.	**1st Qu**.	**Median**	**Mean**	**3rd Qu**.	**Max**.
Heart rate (bpm)	32	54	70	73	89	211
Energy expenditure per day (kcal)	778	1,121	1,293	1,296	1,476	2,576
Steps per day	11	3,982	7,373	8,054	11,530	34,052
Distance covered per day (m)	8	2,747	5 035	5,627	7,933	26,610
Sleep time per day (h)	4	7	8	8	9	18
Temperature (°C)	24	34	35	34	36	44

## Discussion

Overall, acceptance of wearables was high in rural Burkina Faso and seemed to be independent of individual factors like age, sex, and study arm. Accelerometry data were generated most reliably, while photoplethysmography and thermometer measurements proved more difficult with higher data missingness. Data quantity and quality did not appear to be affected by acceptability. Rather, open communication and regular follow-ups of study participants are needed to avoid distress and improper use of the wearables.

### Objective 1: Acceptability—Hindering and enabling factors

Overall, the wearable devices were highly accepted among study participants and field workers. Field workers reported that study participants were enthusiastic, with some describing the wearable as a fashionable item well-taken care of. Resultantly, some wanted to extend their participation in the study. In line with other research in sub-Saharan Africa (15, 16, 46), study participants were interested in their personal health data and showed an overall openness to this new technology. If study participants had better access to their wearable health data, for example, with training showing them how to access this data, this interest may even be increased. Also, study participants in our study were quite young, with a median age of 26 years, which may explain why there was such a high level of acceptance for new technology since younger people are typically more responsive and adaptable to new technologies. This may also highlight the potential for future global health research to use wearables, as nearly half of Burkina Faso's population is under 14 years (45%) (47). Another aspect that may have contributed to high acceptance may be social desirability. About 44% of Burkinabés live on <$1.90 per day (47). The compensation of US $6, which reflects a typical participation rate in the Nouna HDSS, may have been considerable for some and boosted acceptance. Participants may not have provided negative responses for fear of losing their study compensation or being excluded from future study participations. This bias in a such low-resource setting is well-documented (48, 49).

Few participants considered possible adverse health effects of wearables. Some peers mistook the wearables for monitoring devices for HIV- or COVID-19-positive status, raising concerns about stigmatization. Field workers shared with us that study participants felt worried about who could access their data. The presence of terrorism in Burkina Faso is likely to have sensitized the population to such matters. Other studies using wearables in sub-Saharan Africa also reported similar concerns about adverse health effects, stigmatization, and data security (15, 16). Our study's wearables did not capture GPS data or any other personally identifiable information. Data protection for participants must be a top priority.

Reasons that may have impeded wearing the wearable may be derived from cultural factors, as some questionnaire responses suggested that familial acceptance, especially of the household head, may have an impact on young or female study participants' compliance and study participation. In Burkina Faso, most households are led by male household heads, like husbands, fathers, or other male family members (50). Considering that decision-making and risk assessment are more of a familial than an individual issue (50), the participation of female study participants and/or children may have been hindered as a result. The social environment outside the family may also have a substantial impact on study involvement and adherence, especially in rural areas of Africa, where community bonds are especially strong (51). Thus, it appears that not only the acceptability of the study participants, but also that of their family and community members, is crucial for the effective implementation of wearables as a routine monitoring instrument in such settings. Therefore, previous involvement with the community by study managers and/or field workers who introduce the study, the device, and its necessity and advantages for the community appears to be a prerequisite for introducing such novel devices.

We also discovered that members of the family or community associated wearables with magic, HIV- or COVID-19-positive status (surveillance), and adverse health effects, which may also hinder study participation. In our experience, such aspects may be mitigated through regular and transparent communication, explaining the study and wearable devices to study participants, household heads, and community leaders from the beginning (community engagement). Support and question-and-answer sessions for study participants on a regular basis may boost their involvement and compliance.

Participants rarely reported itching, excessive sweating, or perceiving the wearable as generally disturbing. Participants were advised to tighten the wearable wristband, which may have been perceived as too tight by some participants who may have loosened the wristband resulting in heart rate measurement failures. We trained field staff to regularly check the correct position of the wearable and tightness of the wristband, which eventually translated into an increase in data coverage. The number of valid measurements has increased as a result of providing participants with clear instructions on how to wear the device and conducting regular follow-ups on the correct usage.

### Objective 2: Quantity and quality of wearable data to understand individual sleep, activity, and heart rate characteristics within vulnerable populations

During the study, six devices were damaged, which was in the scope of our expectations. Other studies have reported on damaged or lost wearables, corroborating our findings (52) (two WPHR, 4%; four thermometer patches, 17%). Heat and dust exposures in the study environment may have contributed to the deterioration of wearables, leading to inaccurate or missing measurements. Given that 69% of Burkina Faso's population live in rural areas and are mostly engaged in subsistence agriculture (47), wearables are exposed to dust, heat and persistent, long-term physical stress as people engage in farming (intensive manual work combined with soil contact).

Overall, the amount of data acquired for each participant ranged from no data to nearly a complete dataset containing all three variables of physical activity (steps), sleep, and heart rate. In comparison to other studies, it appears as though we were able to obtain a higher amount of data than reported elsewhere (16).

#### Wearable data quality: Individual sleep, activity, and heart rate

As we summarized the data (see Table 6, Supplementary material S3D), we found that some measurements were clearly outliers (i.e., heart rate of 32 bpm). However, it was not entirely clear to us, as even more extreme levels of activity, such as the physical activity of 34,000 steps/day, may reflect the high physical activity level of rural Burkinabé and may not be considered an anomaly in this context, although it would be in other contexts, like urban ones or high-income contexts. Other studies undertaken in sub-Saharan Africa have revealed high levels of physical activity among young rural populations and relatively low levels among urban populations (11, 53, 54). Therefore, it has to be considered that regional and age factors likely contribute to physical activity variances (55). We observed a high number of body shell temperature measurements outside of a normal range of 34–37°C (see Supplementary material S3D) (56). Likely, the patch recorded ambient room temperature when participants did not wear the device. For future analysis, we may only use values within the normal body temperature range and included possibly feverish readings up to 41°C.

Interestingly, we found that the participants' activity levels decreased in the afternoon (Figure 8), possibly as a result of heat stress, which generally peaks during these hours. Thus, wearable data may reveal the direct effect of heat on an individual level in vulnerable populations, which may allow for future studies focusing on climate change-induced weather extremes and health that help to understand individual exposures. As climate change is expected to have a significant impact on sub-Saharan African countries, particularly in the form of increased heat exposures with adverse health and nutritional security impacts, there is a pressing need for adaptation strategies (57, 58). However, there is a dearth of data to inform research and decision-making, and technological innovations such as wearables can make a substantial contribution, as we have established in this study that wearables can create relevant data in low-resource contexts. In that way, interventions could be better targeted and tailored for public health and prevention interventions in Burkina Faso, and likely in similar countries in the Southern African region. Long-term, we anticipate that this type of data collection will support improved prevention and public health approaches that are more appealing to those who directly benefit, as the data can be collected more efficiently and cost-effectively, while also providing individual-level data that can help us to understand distinct subpopulations in terms of climate change and health exposures, but also in terms of the larger picture of communicable and non-communicable diseases. Our study is one of the first to investigate wearables in low-resource, rural communities; therefore, additional research is required to better understand barriers, facilitators, best practices, and how to increase benefits for research and decision-making (59).

#### Data coverage and influencing factors

Generally, data completeness was rather low; however, other studies using wearables even experienced higher levels of missing data (16, 60–63) as we encountered in our study. In a number of villages in the study area, intermittent internet connection prevented data synchronization on-site, necessitating the return of wearables to the Nouna HDSS center (CRSN). This procedure also contributed to missing data, as wearables were not worn by study participants for a couple of days. In addition, data completeness is contingent upon its underlying definition. Other studies, for instance, handled missing data differently and allowed longer periods before declaring data as missing (16, 60–63). For example, another study in SSA using accelerometry sensors to assess epilepsy reported a median of 30% data coverage, whereby a day was reported as 100% data coverage when more than 4 h of data per day were available (16). Currently, there is no standard for reporting the completeness of wearable data making comparison difficult. Furthermore, Kruizinga et al. (61) emphasized that even with some data missing, wearable-generated datasets are substantially larger than non-wearable studies, which may outweigh the disadvantages of missing data, and the pursuit of achieving 100% data completeness. Although most study participants reported to have worn the wearables continuously, several have been cautious fearing breaking the wearables and having to pay for them. Other studies observed similar concerns (16). As a precaution, participants may have taken off the wearables, resulting in missing data. It may be advisable to encourage study participants to take good care of the wearable and emphasize that any accidental damage will not be charged to them.

Age, sex, acceptability, or even dislike of wearables had little effect on data coverage. Mainly, technical issues caused low data coverage, rather than non-compliance of participants, although individual factors may still have an influence. Data coverage of activity data declined over time, possibly due to data synchronization issues. Even when a mobile data connection was established, data were not always sent to the cloud. Furthermore, neither of the two wearable device suppliers offered platforms for manual data download or raw data access. For this study, we used the consumer-focused platforms for data synchronization and study participant management, which may not have been fit to host a larger number of wearable users, as it was the case in our study.

Regarding the thermometer data, participants and field staff reported that the r patch did not adhere to the skin for longer periods and did not connect *via* Bluetooth or synchronized with tablets used by field workers to collect data. To resolve this issue, we had to update all Android-based smartphones to the latest version. Other studies also reported on connectivity issues (16, 46, 63). During the study, we have sensitized field workers to monitor data synchronization closely and fixated the thermometer patch with an adhesive tape to keep it from falling off.

Regarding data of the wristband wearable, both, activity (steps) and heart rate data, were collected by the same device, but with two different sensors (accelerometer and photoplethysmography), yet there were large differences in data coverage. Other research (61), as well as user forums and articles (64–67), reported on discrepancies between the two measurements, and a high level of missing heart rate data seems not uncommon for such devices. Heart rate measurements may be particularly vulnerable and affected by high transpiration (due to the climate with rather extreme heat periods), dark skin (photoplethysmography may not have penetrated the skin sufficiently), improper sensor positioning (as some consumer-grade devices have a single, small photoplethysmography sensor), loose wristbands, and intense movement (17, 18, 68). The quality of heart rate data is rarely studied despite the abundance of validation and accuracy research (3, 69, 70). More research is needed to understand how diverse factors effect heart rate measurements in different populations, such as in rural, low-resource settings.

### Limitations

We acknowledge that reported acceptability was high and may have been influenced by social desirability. Also, data completeness was low, thus decreasing our confidence in drawing conclusions. Furthermore, possible effects of acceptability, sex, or age might not have been identified. Additionally, wearable measurements may be inaccurate and invalid to some extent. Data provided by wearables are not standardized but differ from wearable brand's algorithms and internal data processing. Furthermore, the amount of missing data may have influenced our characterizations of sleep, activity, heart rate, and body shell temperature. However, as this is a case study which primarily focused on understanding general acceptability, reliability, and feasibility of wearable devices, the primary outcome was not statistical validation but technical and human factors, as well as identifying barriers and the feasibility of collecting data with wearables and their acceptability (7).

As the output data of both wearables are preprocessed by the manufacturer (details on data preprocessing are undisclosed), single output values may thus be aggregated values. We did not calculate data coverage for WPHR sleep data since the aforementioned approach (defining 8 h as sleep length) has some imprecision and sleep diaries would be inadequate in our context. Because the WPHR uses accelerometry to track sleep, the data coverage of the activity data served as an estimate.

It has to be noted that there is no standardized, established method to analyze missing data of consumer-grade wearables. Utilizing tolerance limits (60/5/15 min) for data coverage, we have established a method that we deemed appropriate for our study based on exchanges with experts and comprehensive literature analysis. These tolerance limits might influence insights on data coverage. However, we explored different limits to understand the impact on outcomes of data coverage and found no relevant differences in tolerance limits (see Supplementary material S3A).

## Conclusion

Overall, both wearable devices—wrist-worn wearable and thermometer patch—were highly accepted by study participants in the Nouna HDSS in Burkina Faso. Study participants showed interest and desire for both wearables, and few expressed concerns or reported adverse health effects. Data completeness was higher than previously reported, but we still encountered data missingness, particularly in heart rate and temperature measurements which warrants further research. Nevertheless, with both wearables, we were able to generate continuous datasets incorporating individual-level activity and vital data. We found several major criteria for data missingness to be mostly technical in nature, including damaged wearable devices—also likely caused by prolonged exposures to dust and heat—intermittent data synchronization, and Bluetooth connectivity.

As the epidemiological transition progresses throughout sub-Saharan Africa, life lived with diseases is an increasingly important part of a population's burden of disease, particularly for climate change vulnerable, low-resource countries such as Burkina Faso.

In rural, low-resource contexts, wearables may for the first time provide objective insights into the activity and vital patterns of the individuals. Our study underlines that wearables can generate large and longitudinal datasets on activity (steps), sleep duration and quality, and heart rate, which can be added to existing population health routine measurements such as in the HDSSs. This could be crucial for designing and targeting public health and behavior change interventions in LMICs. To our knowledge, this is the first study to use consumer-focused wearables to generate individual data in the rural setting of Burkina Faso. Future research may aim to explore factors hindering heart rate measurements, especially in non-laboratory settings, as well as barriers and facilitators for using wearables for population health surveillance in LMICs.

## Data availability statement

The data sets generated during the study are not publicly available due to the small sample size and concerns regarding participant confidentiality and anonymity but may be made available in a deidentified version from the corresponding author upon request.

## Ethics statement

Ethical approval for this study was provided by the Ethics Committee of the Medical Faculty, Heidelberg University, in 21/05/2019 (S-294/2019), and the Comité d'ethique pour la recherche en santé in Burkina Faso (approval date: 13th March, 2020; 2020-3-041). Written informed consent to participate in this study was provided by the participants' legal guardian/next of kin. Written informed consent was obtained from the individual(s), and minor(s)' legal guardian/next of kin, for the publication of any potentially identifiable images or data included in this article.

## Author contributions

SB, TB, and AS conceived and designed the project. AS, VB, and WAO managed the study in Burkina Faso with remote guidance of SB, SH, AB, MAM, and H-CG. SH monitored the data during the study, analyzed the data, and drafted the manuscript in close collaboration with SB. All authors contributed to the critical revision of the draft and approved the final version of the manuscript.

## Funding

We wish to thank the German Research Foundation (DFG) for supporting this study as part of the FOR Climate Change and Health in Sub-Saharan Africa. The German Research Foundation (DFG) is supporting this study, but has not been involved in study design, collection, management, analysis, or interpretation of data, neither in the writing of this report nor in any decision to submit this report for publication. The study has been approved by DFG (FOR 2936/project: 427397328).

## Conflict of interest

The authors declare that the research was conducted in the absence of any commercial or financial relationships that could be construed as a potential conflict of interest.

## Publisher's note

All claims expressed in this article are solely those of the authors and do not necessarily represent those of their affiliated organizations, or those of the publisher, the editors and the reviewers. Any product that may be evaluated in this article, or claim that may be made by its manufacturer, is not guaranteed or endorsed by the publisher.

## Supplementary material

Supplementary material S1 Consolidated Standards of Reporting Trials (CONSORT) extension for randomized pilot and feasibility trials checklist.

Supplementary material S2 Original questionnaire.

Supplementary material S3 Supplementary tables and figures concerning A: analysis of data completeness calculation, B: acceptability, C: data completeness, and D: wearable measurements.

## Abbreviations

MC: multiple choice (questions)
MEMS: micro-electromechanical system
Tucky: Tucky axillary thermometer patch
Wearable: consumer-grade Wearable device
WPHR or Withings: Withings Pulse HR fitness tracker wristband.
