# Supplementary material for: Using wearable devices to generate real-world, individual-level data in rural, low-resource contexts in Burkina Faso, Africa: A case study
Source: Front Public Health. 2022 Sep 30;10:972177. doi: 10.3389/fpubh.2022.972177 (PMC9561896; doi:10.3389/fpubh.2022.972177)
Supplement: Supplementary material S1 — Consolidated Standards of Reporting Trials (CONSORT) extension for randomized pilot and feasibility trials checklist. [file Table_1.DOCX]

Supplementary Material

# Further analysis of data coverage calculation.

For the following figures in the appendix, we calculated the mean of Likert-type responses. We are aware that some researchers advocate against doing so. However, we used this approach as we found it adequate to visualize responses in a comprehensive way and not to dilute statistical findings.


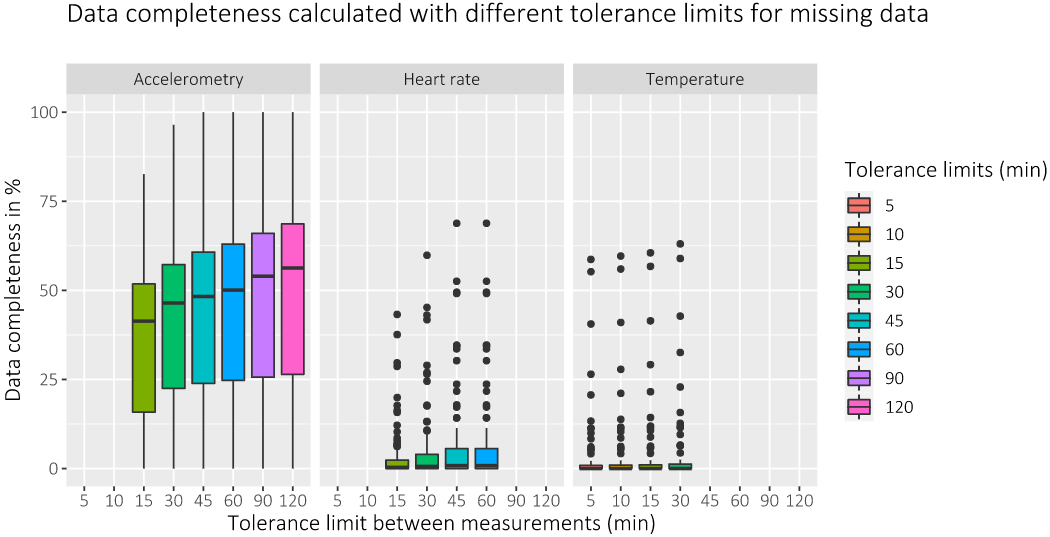


**Figure 1.** Data coverage calculated with different tolerance limits of time differences between measurements. Thus, the tolerance limits are the highest interval between two measurements not seen as missing data but tolerated as technical variability. Only reasonable limits regarding the respective sampling rate are displayed, not all possible limits. As help to evaluate the data coverage analysis.


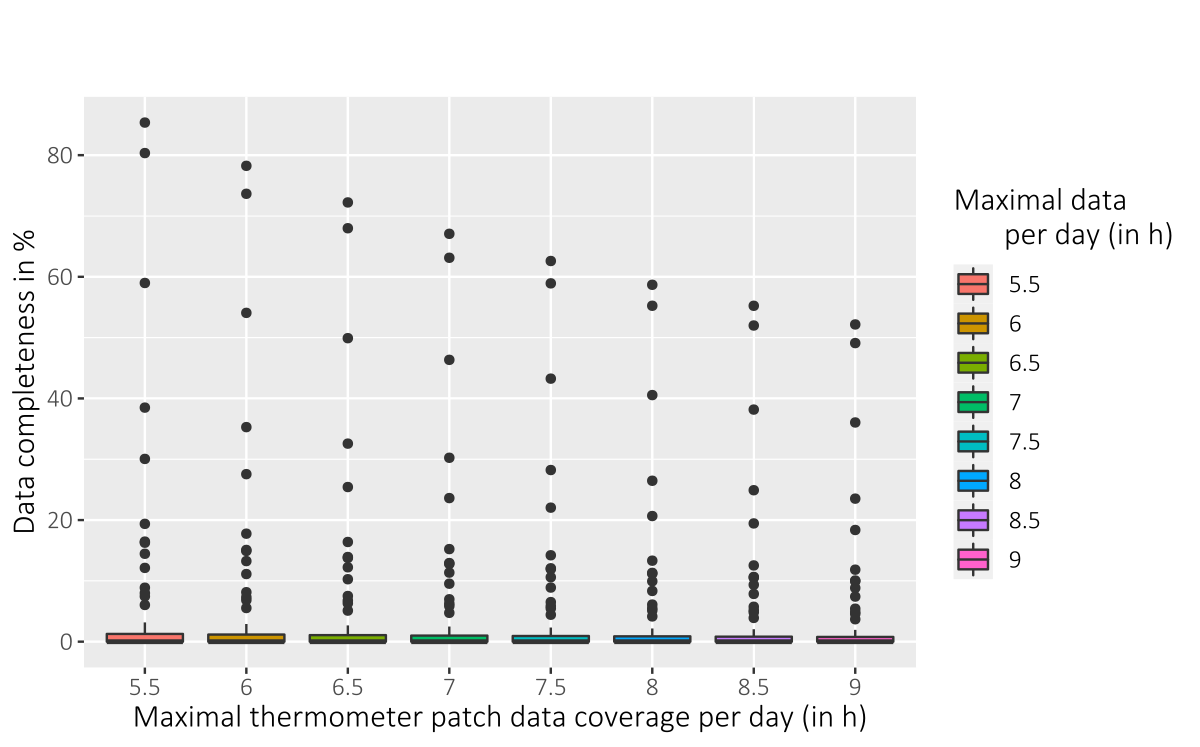


**Figure 2.** Data coverage of body shell temperature measurements calculated with different values for maximal data coverage per day (i.e., maximal or rather typical sleep duration). As help to evaluate the data coverage analysis for thermometer patch data.

# Acceptability of subgroups of sex, age, study arm and cycle; negative questionnaire responses per cycle and sex.

**
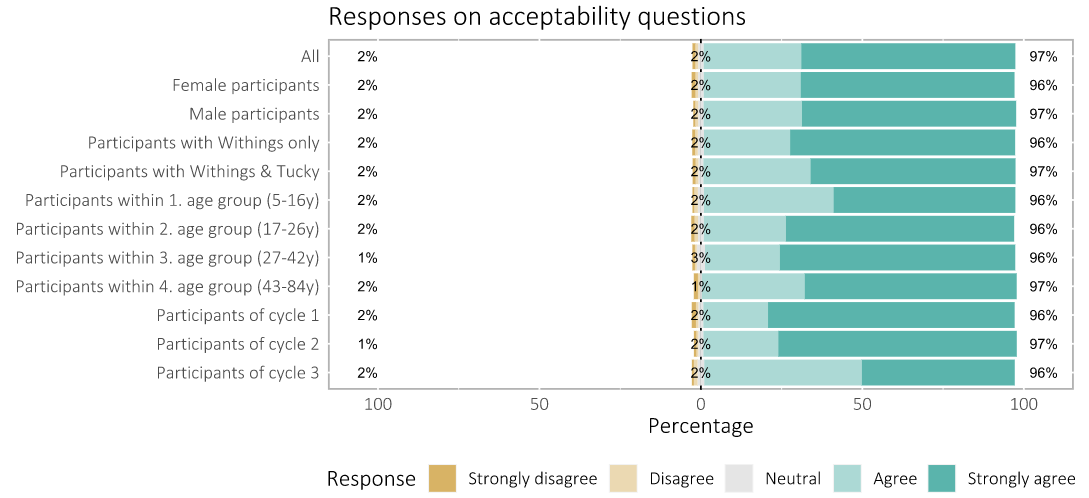
**

**Figure 3. Total sum of all Likert-scaled questionnaire responses (n=11 872) across all items concerning Acceptability (n=14). The sums of each Likert-scale-level are displayed as total and according to participant’s sex, study arm, age group and study cycle (see each line/bar).**


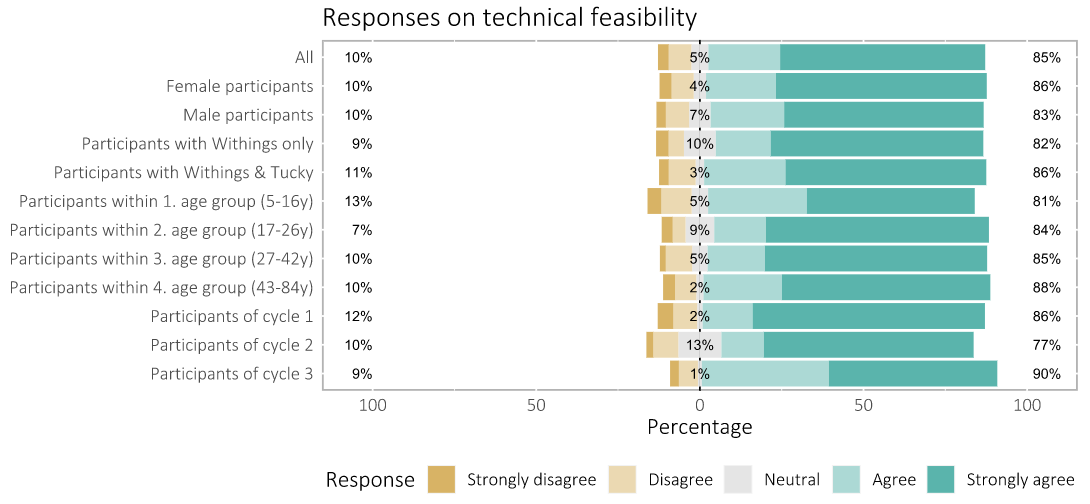

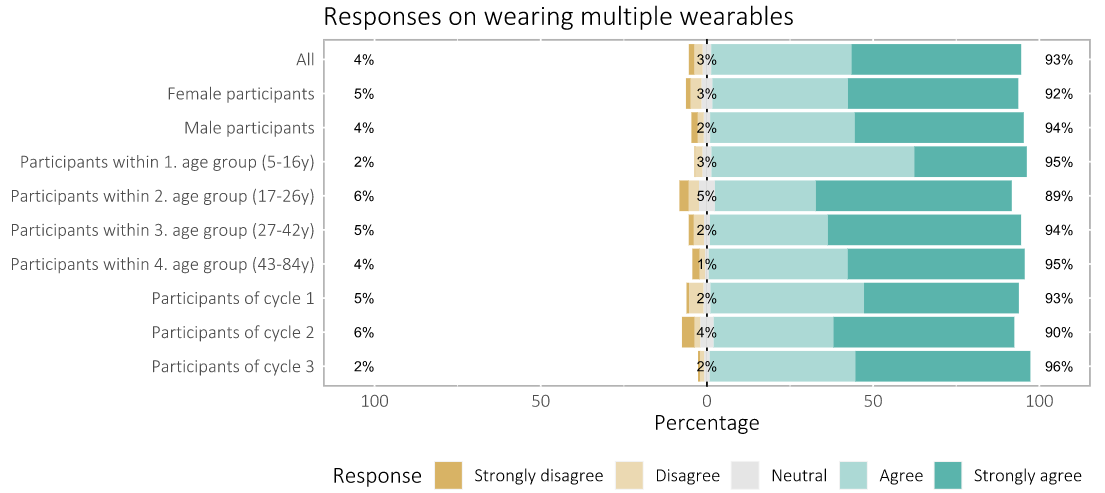


**Figure 4. Sums of all Likert-scaled questionnaire responses (n=3 392) across items concerning wearing multiple wearables (n=4)** answered by participants; as well as s**ums of all Likert-scaled questionnaire responses (n=2 544) across items technical feasibility (n=3)** answered by field workers**. The sums of each Likert-scale-level are displayed as total and according to participant’s sex, study arm, age group and study cycle (see each line/bar).**


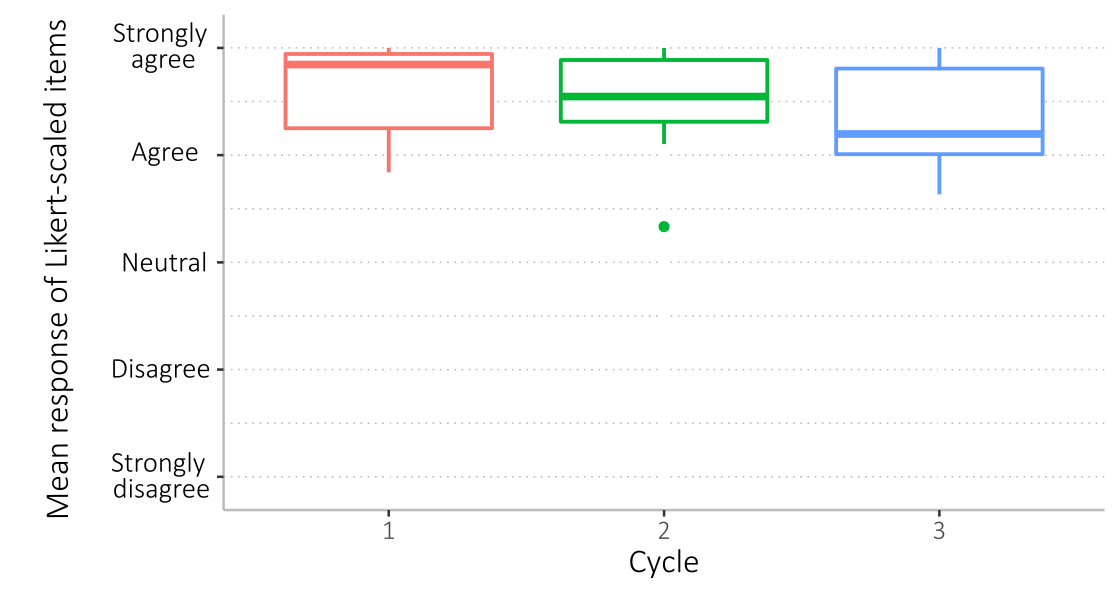


**Figure 5**. Average responses on Likert-scaled question items for all study participants per study cycle.

# Further data quality and quantity analysis: Data completeness of subgroups of sex, age, study arm, and data completeness in relation to negative questionnaire responses.


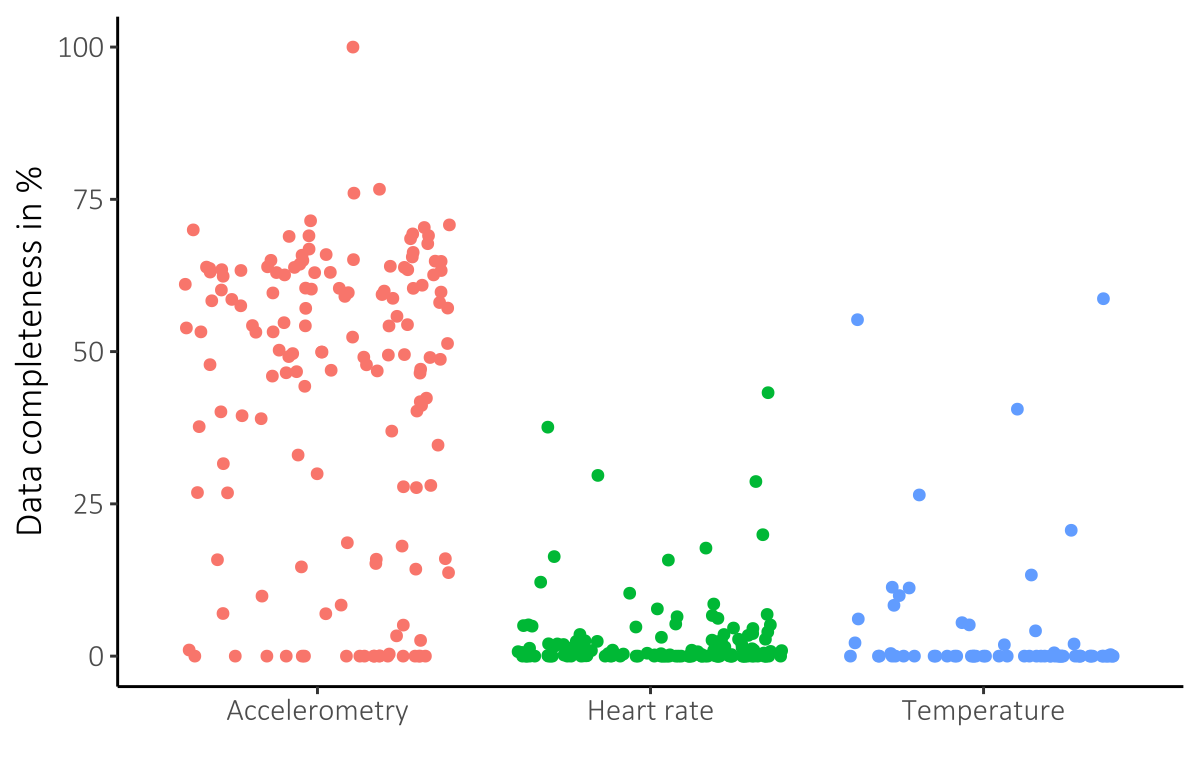


**Figure 6**. Overview of data completeness of each study participant for the variables of accelerometery, heart rate and body shell temperature, for the complete 9-week study period.


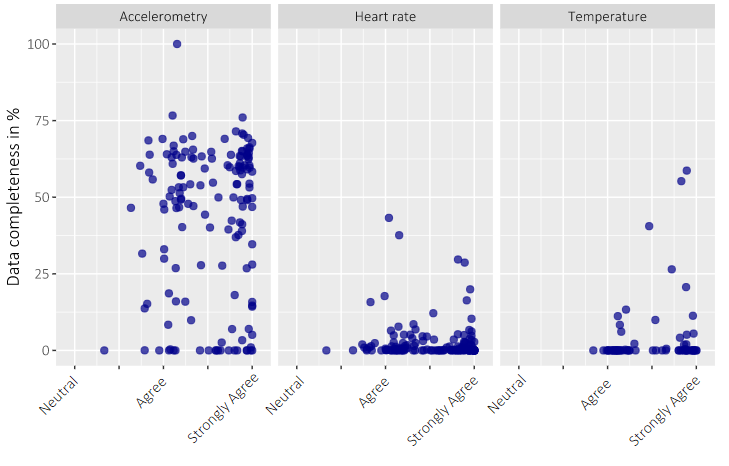
**Figure 7**. Data completeness in relation to participant’s average questionnaire responses. For calculating the average questionnaire response, Likert-scaled responses were recoded (five for “Strongly Agree”, four for “Agree”, three for “Neutral”, etc.) and the mean calculated for every study participant.

**
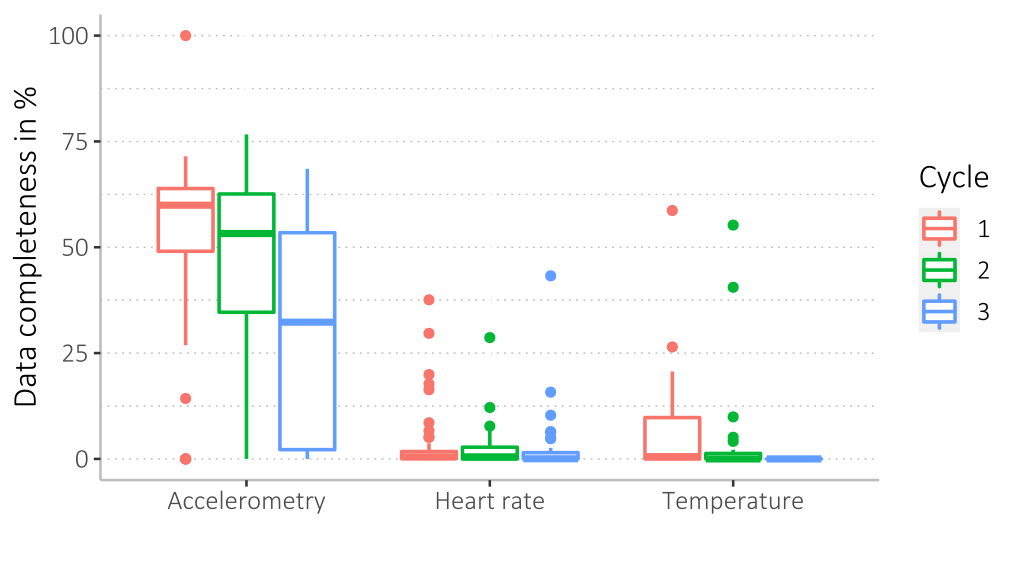
**

**Figure 8**. Data completeness for the variables of accelerometery, heart rate and body shell temperature data per the three study cycles, covering all study participants during the complete 9-week study period.

# Wearable measurement overview


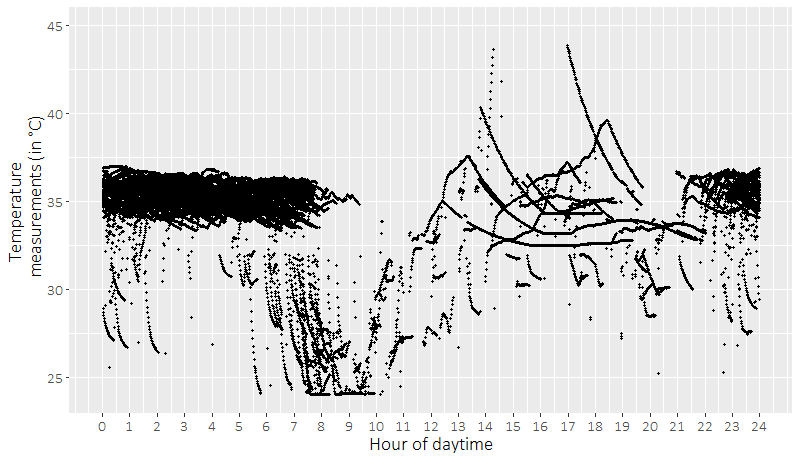


**Figure 9**. Temperature measurements Overview of all body shell temperature measurements taken during the day of all participants as measured by the Tucky thermometer wearable patch.

**
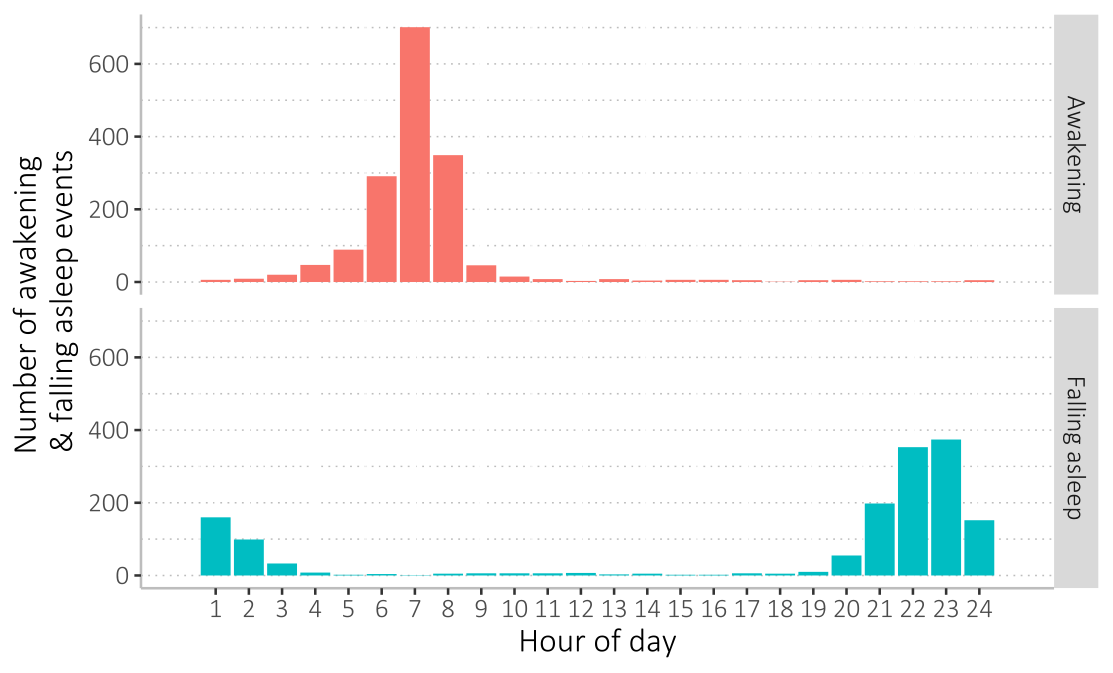
**

**Figure 10.** Number of awakening and falling asleep events during the day. Measured with the WPHR, for all study participants over the full 9-week study period. Most awakenings happened around 7am, most falling asleep events in the late evening and early night (around 21:00-24:00).
