# Supplementary material for: Using wearable devices to generate real-world, individual-level data in rural, low-resource contexts in Burkina Faso, Africa: A case study
Source: Front Public Health. 2022 Sep 30;10:972177. doi: 10.3389/fpubh.2022.972177 (PMC9561896; doi:10.3389/fpubh.2022.972177)
Supplement: Supplementary material S3 — Supplementary tables and figures concerning A: analysis of data completeness calculation, B: acceptability, C: data completeness, and D: wearable measurements. [file Data_Sheet_2.PDF]

Generated by vboudo, Jan 14, 2021 12:58
Questionnaire created by sandrabarteit, Oct 23, 2020 14:48
Last modified by vboudo, Jan 14, 2021 12:58

Shared with:
vboudo last edited 1/14/2021 12:58:41 PM
WARISTIDE (never edited)
S_Huhn (never edited)
MaggioniMA (never edited)

Sections: 4, Sub-sections: 0, Questions: 66.
Questions with enabling conditions: 14
Questions with validation conditions:0
Rosters: 2
Variables: 0

Survey Solutions

CP1 Étude de faisabilité

SURVEY IDENTIFICATION INFORMATION
QUESTIONNAIRE DESCRIPTION

DÉTAILS DES PARTICIPANTS À L'ÉTUDE
No sub-sections, No rosters, Questions: 2, Static texts: 1.

DISTRIBUTION/RETOUR DE CAPTEUR
No sub-sections, Rosters: 2, Questions: 28.

QUESTIONNAIRE D'ACCEPTATION
No sub-sections, No rosters, Questions: 29, Static texts: 1.

JOURNAL D'ACTIVITÉ
No sub-sections, No rosters, Questions: 7.

APPENDIX A — CATEGORIES

LEGEND

SURVEY IDENTIFICATION INFORMATION QUESTIONNAIRE DESCRIPTION

Basic information

Title CP1 Étude de faisabilité

Survey data information

Study type Integrated Survey

Kind of data Aggregate data [agg]

Survey information

Country Burkina Faso

Year 2020

Languages French

Funding DFG

DÉTAILS DES PARTICIPANTS À L'ÉTUDE

study_part_details

STATIC TEXT

Procédure d'étude: --- 1. Prenez le capteur du participant --- --- 2. Désinfectez le capteur du participant --- --- 3. Mettez le capteur sur le chargeur --- --- 4. Synchronisez les données du capteur avec le profil du participant à l'étude avec l'app en la tablette.--- --- 5. Remplir la section "Distribution/retour de capteur" --- --- 6. Prenez la mesure de la pression artérielle du participant et enregistrez-le dans l'application Health Mate --- --- 7. Posez les questions du questionnaire d'acceptation --- --- 8. Posez les questions du questionnaire de journal d'activité ---

Saisissez l'ID du participant à l'étude<br><br>I L'identifiant du participant est de 14 position .	TEXT study_part_id<br><br>..
Nom du village du participant	SINGLE-SELECT: COMBO BOX nom_village<br>001 ○ Barakuy<br>002 ○ Toni<br>003 ○ Biron Bobo<br>004 ○ Biron Marka<br>005 ○ Boron<br>006 ○ Bouni/Boune<br>007 ○ Bourasso<br>008 ○ Cisse<br>009 ○ Dankoumana<br>010 ○ Dembelela<br>011 ○ Denissa<br>012 ○ Denissa Mossi<br>013 ○ Diamasso<br>014 ○ Dionkongo<br>015 ○ Dina<br>016 ○ Dokoura<br><br>And 272 other symbols [4]

DISTRIBUTION/RETOUR DE CAPTEUR

sensor_exchange

Question	Réponse
Voulez-vous distribuer ou retourner un capteur?	SINGLE-SELECT — return_handout_sensor<br>01 ○ Distribuer - Je voudrais commencer la distribution d'un capteur.<br>02 ○ Retourner - Je voudrais commencer le retour d'un capteur.
Quel capteur est distribué?<br>E return_handout_sensor==1	MULTI-SELECT — sensor_type_distrib<br>01 ☐ Withings Pulse HR (noir poignet)<br>02 ☐ Tucky thermomètre (blanc tache)<br>03 ☐ Aucune

DISTRIBUTION/RETOUR DE CAPTEUR

Roster: QUEL CAPTEUR EST DISTRIBUÉ? - %ROSTERTITLE%

generated by multi-select question sensor_type_distrib

roster_1_sensor_distributed

E return_handout_sensor==1

Question	Réponse
Quel numéro a le capteur %rostertitle% distribué?	NUMERIC: INTEGER — num_sensor_distributed<br>_ .
Veuillez noter la date de distribution du capteur %rostertitle%.<br>I Veuillez remplir la date sous la forme jour (jj) - mois (mm) - année (aa)	DATE — sensor_date_distributed<br>..
Le capteur %rostertitle% est-il désinfecté?	SINGLE-SELECT — sensor_desinfected<br>01 ○ Oui, il est désinfecté.<br>02 ○ No, il n'est pas désinfecté.<br>03 ○ Commentaire
Commentaire (desinfection de capteur)<br>E sensor_desinfected==3	TEXT — sensor_desinfected_comm<br>..
Le participant à l'étude est enregistré dans l'application sur la tablette pour le capteur %rostertitle%?	SINGLE-SELECT — registration_stud_part_app<br>01 ○ Oui, j'ai inscrit le participant à l'étude dans l'application<br>02 ○ Non, je n'ai pas enregistré le participant à l'étude dans l'application<br>03 ○ Commentaire
Commentaire (enregistrement)<br>E registration_stud_part_app==3	TEXT — registration_stud_part_app_comm<br>..
Est ce que vous etes à l'aise avec l'application pour la tablette du capteur %rostertitle% ?	SINGLE-SELECT — ease_app_distribution<br>01 ○ Tout à fait d'accord<br>02 ○ D'accord<br>03 ○ Ni en désaccord ni d'accord<br>04 ○ Pas d'accord<br>05 ○ Pas du tout d'accord

était il facile pour vous de synchroniser les données du capteur du participant à l'étude.	SINGLE-SELECT difficulty_usage_app_distributi<br>01 ◯ Tout à fait d'accord<br>02 ◯ D'accord<br>03 ◯ Ni en désaccord ni d'accord<br>04 ◯ Pas d'accord<br>05 ◯ Pas du tout d'accord
Il était facile pour vous de gérer le participant à l'étude avec la tablette.	SINGLE-SELECT knowledge_level_app_distribution<br>01 ◯ Tout à fait d'accord<br>02 ◯ D'accord<br>03 ◯ Ni en désaccord ni d'accord<br>04 ◯ Pas d'accord<br>05 ◯ Pas du tout d'accord
Le participant est satisfait du capteur %rostertitle%.	SINGLE-SELECT feedback_stud_part_distribution<br>01 ◯ Tout à fait d'accord<br>02 ◯ D'accord<br>03 ◯ Ni en désaccord ni d'accord<br>04 ◯ Pas d'accord<br>05 ◯ Pas du tout d'accord
Le participant accepte le capteur %rostertitle%.	SINGLE-SELECT acceptance_stud_part_distributio<br>01 ◯ Tout à fait d'accord<br>02 ◯ D'accord<br>03 ◯ Ni en désaccord ni d'accord<br>04 ◯ Pas d'accord<br>05 ◯ Pas du tout d'accord
Le participant était heureux de porter le capteur %rostertitle%.	SINGLE-SELECT happy_stud_part_distribution<br>01 ◯ Tout à fait d'accord<br>02 ◯ D'accord<br>03 ◯ Ni en désaccord ni d'accord<br>04 ◯ Pas d'accord<br>05 ◯ Pas du tout d'accord
Le participant est frustré par le capteur %rostertitle%.	SINGLE-SELECT frustration_stud_part_distributi<br>01 ◯ Tout à fait d'accord<br>02 ◯ D'accord<br>03 ◯ Ni en désaccord ni d'accord<br>04 ◯ Pas d'accord<br>05 ◯ Pas du tout d'accord
Quel capteur est retourné?<br>E return_handout_sensor==2	MULTI-SELECT sensor_type_returned<br>01 ☐ Withings Pulse HR (noir poignet)<br>02 ☐ Tucky thermomètre (blanc tache)<br>03 ☐ Aucune

DISTRIBUTION/RETOUR DE CAPTEUR

Roster: QUEL CAPTEUR EST RETOURNÉ? - %ROSTERTITLE%

generated by multi-select question sensor_type_returned roster_2_sensor_returned

E return_handout_sensor==2

Question	Response	Variable
Quel numéro a le capteur %rostertitle% retourné?	NUMERIC: INTEGER<br>______________________	num_sensor_returned
Veuillez noter la date de retour du capteur %rostertitle%.<br>I Veuillez remplir la date sous la forme jour (jj) - mois (mm) - année (aa)	DATE<br>______________________	sensor_date_returned
Le capteur %rostertitle% est-il défectueux?	SINGLE-SELECT<br>01 ○ Oui, défectueuse<br>02 ○ No, travail<br>03 ○ Autre	sensor_status_returned
Autre (capteur %rostertitle% défectueux)<br>E sensor_status_returned==3	TEXT<br>______________________	comment_sensor_status_returned
Etes vous à l'aise avec l'application pour la tablette du capteur %rostertitle% ?	SINGLE-SELECT<br>01 ○ Tout à fait d'accord<br>02 ○ D'accord<br>03 ○ Ni en désaccord ni d'accord<br>04 ○ Pas d'accord<br>05 ○ Pas du tout d'accord	ease_usage_app
Il était facile pour vous de synchroniser les données du capteur du participant à l'étude.	SINGLE-SELECT<br>01 ○ Tout à fait d'accord<br>02 ○ D'accord<br>03 ○ Ni en désaccord ni d'accord<br>04 ○ Pas d'accord<br>05 ○ Pas du tout d'accord	difficulty_usage_app
Il était facile pour vous de gérer le participant à l'étude avec la tablette.	SINGLE-SELECT<br>01 ○ Tout à fait d'accord<br>02 ○ D'accord<br>03 ○ Ni en désaccord ni d'accord<br>04 ○ Pas d'accord<br>05 ○ Pas du tout d'accord	knowledge_level_app
Le participant est satisfait du capteur %rostertitle%.	SINGLE-SELECT<br>01 ○ Tout à fait d'accord<br>02 ○ D'accord<br>03 ○ Ni en désaccord ni d'accord<br>04 ○ Pas d'accord<br>05 ○ Pas du tout d'accord	feedback_stud_part
Le participant accepte le capteur %rostertitle%.	SINGLE-SELECT<br>01 ○ Tout à fait d'accord<br>02 ○ D'accord<br>03 ○ Ni en désaccord ni d'accord<br>04 ○ Pas d'accord<br>05 ○ Pas du tout d'accord	acceptance_stud_part

Le participant était heureux de porter le capteur %rostertitle%.	SINGLE-SELECT happy_stud_part<br>01 ○ Tout à fait d'accord<br>02 ○ D'accord<br>03 ○ Ni en désaccord ni d'accord<br>04 ○ Pas d'accord<br>05 ○ Pas du tout d'accord
Le participant est frustré par le capteur %rostertitle%.	SINGLE-SELECT frustration_stud_part<br>01 ○ Tout à fait d'accord<br>02 ○ D'accord<br>03 ○ Ni en désaccord ni d'accord<br>04 ○ Pas d'accord<br>05 ○ Pas du tout d'accord
Autres commentaires	TEXT general_comments_sensor_return<br>..

QUESTIONNAIRE D'ACCEPTATION

STATIC TEXT

Avant de commencer l'entretien avec le participant à l'étude, veuillez mettre le (s) capteur (s) sur le chargeur. Veuillez poser toutes les questions au participant à l'étude. Merci pour votre précieuse collaboration.

Je suis content d'utiliser le capteur.	SINGLE-SELECT `positive_att_sensor`<br>01 ○ Tout à fait d'accord<br>02 ○ D'accord<br>03 ○ Ni en désaccord ni d'accord<br>04 ○ Pas d'accord<br>05 ○ Pas du tout d'accord
Je suis dérangé lors de l'utilisation du capteur.	SINGLE-SELECT `disturbed_sensor`<br>01 ○ Tout à fait d'accord<br>02 ○ D'accord<br>03 ○ Ni en désaccord ni d'accord<br>04 ○ Pas d'accord<br>05 ○ Pas du tout d'accord
Portez-vous plus d'un capteur?	SINGLE-SELECT `multiple_sensors`<br>01 ○ oui<br>02 ○ non
Je me sentais à l'aise avec de nombreux capteurs.<br>E `multiple_sensors == 1`	SINGLE-SELECT `experience1_multiple_sensors`<br>01 ○ Tout à fait d'accord<br>02 ○ D'accord<br>03 ○ Ni en désaccord ni d'accord<br>04 ○ Pas d'accord<br>05 ○ Pas du tout d'accord
C'était difficile pour moi de porter de nombreux capteurs.<br>E `multiple_sensors==1`	SINGLE-SELECT `experience2_multiple_sensors`<br>01 ○ Tout à fait d'accord<br>02 ○ D'accord<br>03 ○ Ni en désaccord ni d'accord<br>04 ○ Pas d'accord<br>05 ○ Pas du tout d'accord
C'était ennuyeux pour moi de porter de nombreux capteurs.<br>E `multiple_sensors==1`	SINGLE-SELECT `experience3_multiple_sensors`<br>01 ○ Tout à fait d'accord<br>02 ○ D'accord<br>03 ○ Ni en désaccord ni d'accord<br>04 ○ Pas d'accord<br>05 ○ Pas du tout d'accord
J'ai aimé porter de nombreux capteurs.<br>E `multiple_sensors==1`	SINGLE-SELECT `experience4_multiple_sensors`<br>01 ○ Tout à fait d'accord<br>02 ○ D'accord<br>03 ○ Ni en désaccord ni d'accord<br>04 ○ Pas d'accord<br>05 ○ Pas du tout d'accord

C'était facile pour moi de porter le capteur cette semaine.	SINGLE-SELECT week_experience1_sensor<br>01 ○ Tout à fait d'accord<br>02 ○ D'accord<br>03 ○ Ni en désaccord ni d'accord<br>04 ○ Pas d'accord<br>05 ○ Pas du tout d'accord
C'était ennuyeux pour moi de porter le capteur cette semaine.	SINGLE-SELECT week_experience2_sensor<br>01 ○ Tout à fait d'accord<br>02 ○ D'accord<br>03 ○ Ni en désaccord ni d'accord<br>04 ○ Pas d'accord<br>05 ○ Pas du tout d'accord
Qu'est-ce que vous avez aimé / n'a pas aimé dans le capteur? (choix multiples)	MULTI-SELECT: ORDERED like_dislike_sensor<br>01 ☐ facile à porter<br>02 ☐ bon poids<br>03 ☐ bon saisir<br>04 ☐ belle apparence<br>05 ☐ pratique à porter<br>06 ☐ confortable à porter<br>07 ☐ trop volumineux<br>08 ☐ trop lourd<br>09 ☐ trop grand<br>10 ☐ difficile à porter<br>11 ☐ Autre (décrivez s'il vous plait)
Commentaire (aime/n'aime pas) - décrivez s'il vous plait	TEXT like_dislike_sensor_comment<br>..
Avez-vous eu des problèmes avec le capteur cette semaine?	SINGLE-SELECT challenges_sensor<br>01 ○ Oui<br>02 ○ Non
Des problèmes avec le capteur cette semaine. (choix multiples)<br><br>E challenges_sensor==1	MULTI-SELECT: ORDERED challenges_sensor_y<br>01 ☐ démangeaison de la peau<br>02 ☐ démangeaison<br>03 ☐ capteur causant de la douleur<br>04 ☐ limitation des mouvements<br>05 ☐ dérangé pendant le travail<br>06 ☐ dérangé pendant le sommeil<br>07 ☐ routine quotidienne perturbée<br>08 ☐ choc électrique<br>09 ☐ Autre (décrivez s'il vous plait)
Commentaire (problèmes avec capture cette semaine) - décrivez s'il vous plait	TEXT challenges_sensor_yes_comments<br>..
Le port du capteur a affecté mon travail / mon activité quotidienne.	SINGLE-SELECT daily_life_sensor<br>01 ○ Tout à fait d'accord<br>02 ○ D'accord<br>03 ○ Ni en désaccord ni d'accord<br>04 ○ Pas d'accord<br>05 ○ Pas du tout d'accord

Quelle sensation avez vous en portant le capteurs? (choix multiples)	MULTI-SELECT: ORDERED `reactions_sensor`<br>01 ☐ J'ai oublié que je le portais<br>02 ☐ Je n'ai pas été dérangé<br>03 ☐ Parfois difficile<br>04 ☐ Besoin de temps / d'attention<br>05 ☐ J'ai interrompu mes activités plusieurs fois à cause du capteur<br>06 ☐ J'ai dû retirer le capteur<br>07 ☐ Le capteur limitait mes mouvements<br>08 ☐ J'ai eu des douleurs ou des effets indésirables (comme une poussée cutanée, des démangeaisons)<br>09 ☐ Le capteur était collé sur ma peau à cause de la transpiration<br>10 ☐ J'ai ressenti une augmentation de la chaleur à cause du port du capteur<br>11 ☐ Autre (décrivez s'il vous plait)
Commentaire (capteurs d'effets) - décrivez s'il vous plait	TEXT `reactions_sensor_comment`<br>..
Le port de l'appareil a-t-il eu des effets sur votre sommeil?	SINGLE-SELECT `sleep_sensor`<br>01 ○ Oui<br>02 ○ Non
Le port de l'appareil a eu les effets suivants sur mon sommeil... (choix multiples)<br>E `sleep_sensor==1`	MULTI-SELECT: ORDERED `sleep_sensor_2`<br>01 ☐ Je me suis réveillé parfois<br>02 ☐ Je me suis réveillé fréquemment<br>03 ☐ Je n'ai pas pu dormir du tout à cause du capteur<br>04 ☐ Je me sentais fatigué le matin (mauvaise qualité du sommeil)<br>05 ☐ Le capteur est tombé de moi pendant la nuit<br>06 ☐ J'ai ressenti une augmentation de la chaleur à cause du port du capteur<br>07 ☐ Autre (décrives s'il vous plait)
Commentaire (effets de capteur sur mon sommeil) - décrivez s'il vous plait	TEXT `sleep_sensor_3`<br>..
Avez-vous dû retirer l'appareil?	SINGLE-SELECT `removing_sensor`<br>01 ○ Oui<br>02 ○ Non
J'ai retiré le capteur parce que... (choix multiple)<br>E `removing_sensor==1`	MULTI-SELECT: ORDERED, YES/NO `removing_sensor_2`<br>01 ☐ / ☐ c'était limiter mes activités<br>02 ☐ / ☐ J'ai eu des effets indésirables<br>03 ☐ / ☐ Autre (décrives s'il vous plait)
Commentaire (retrait du capteur) - décrivez s'il vous plait	TEXT `removing_sensor_3`<br>..

J'étais à l'aise de porter le capteur en public.	SINGLE-SELECT sensor_public<br>01 ○ Tout à fait d'accord<br>02 ○ D'accord<br>03 ○ Ni en désaccord ni d'accord<br>04 ○ Pas d'accord<br>05 ○ Pas du tout d'accord
Des gens vous ont-ils posé des questions sur le capteur?	SINGLE-SELECT sensor_public_2<br>01 ○ Oui<br>02 ○ Non
Sur quel capteur les gens vous ont-ils posé des questions<br>E sensor_public_2 == 1	MULTI-SELECT sensor_public_2_1<br>01 ☐ Withings Pulse HR (noir poignet)<br>02 ☐ Tucky thermomètre (blanc tache)<br>03 ☐ Aucune
Décrivez s'il vous plait, qu'est-ce que les gens vous ont demandé sur le capteur.<br>E sensor_public_2 == 1	TEXT sensor_public_2_2<br>..
Si vous deviez porter cet appareil pendant une période plus longue (c'est-à-dire pendant un an), quels seraient les obstacles pour vous de participer à une telle étude (choix multiple)	MULTI-SELECT longterm_sensor<br>01 ☐ Durée nécessaire pour porter<br>02 ☐ temps consacré à la participation aux études<br>03 ☐ le capteur ne me donne pas d'informations sur ma santé<br>04 ☐ Effets indésirables<br>05 ☐ Acceptation sociale<br>06 ☐ Perturbation de l'activité quotidienne<br>07 ☐ Perturbation de mon sommeil<br>08 ☐ Perturbation de ma routine d'hygiène personnelle<br>09 ☐ Autre (décrivez s'il vous plait)
Commentaire (étude de capteurs à long terme sur les barrières) - décrivez s'il vous plait	TEXT longterm_sensor_comment<br>..

JOURNAL D'ACTIVITÉ

Quelle activité avez-vous fait après vous être levé ce matin? (choix multiple)	MULTI-SELECT: ORDERED after_gettingup<br>01 ☐ travaillant à la ferme<br>02 ☐ cuisine<br>03 ☐ vendre des produits sur le marché<br>04 ☐ élevage d'animaux<br>05 ☐ prendre soin des enfants<br>06 ☐ prendre soin de son mari / femme<br>07 ☐ prendre soin des membres de la famille<br>08 ☐ va à l'école<br>09 ☐ chercher de l'eau dans le puits<br>10 ☐ récolte<br>11 ☐ travailler sur le terrain<br>12 ☐ je me reposais<br>13 ☐ regarder la télévision<br>14 ☐ aller au bar<br>15 ☐ aller au restaurant<br>16 ☐ faire du sport<br><br>And 5 other symbols [3]
Quelle activité avez-vous pratiquéz le matin? (choix multiple)	MULTI-SELECT morning_activity<br>01 ☐ travaillant à la ferme<br>02 ☐ cuisine<br>03 ☐ vendre des produits sur le marché<br>04 ☐ élevage d'animaux<br>05 ☐ prendre soin des enfants<br>06 ☐ prendre soin de son mari / femme<br>07 ☐ prendre soin des membres de la famille<br>08 ☐ va à l'école<br>09 ☐ chercher de l'eau dans le puits<br>10 ☐ récolte<br>11 ☐ travailler sur le terrain<br>12 ☐ je me reposais<br>13 ☐ regarder la télévision<br>14 ☐ aller au bar<br>15 ☐ aller au restaurant<br>16 ☐ faire du sport<br><br>And 5 other symbols [3]

Quelle activité as-tu pratiquée le midi? (choix multiple)	MULTI-SELECT `noon_activity`<br>01 ☐ travaillant à la ferme<br>02 ☐ cuisine<br>03 ☐ vendre des produits sur le marché<br>04 ☐ élevage d'animaux<br>05 ☐ prendre soin des enfants<br>06 ☐ prendre soin de son mari / femme<br>07 ☐ prendre soin des membres de la famille<br>08 ☐ va à l'école<br>09 ☐ chercher de l'eau dans le puits<br>10 ☐ récolte<br>11 ☐ travailler sur le terrain<br>12 ☐ je me reposais<br>13 ☐ regarder la télévision<br>14 ☐ aller au bar<br>15 ☐ aller au restaurant<br>16 ☐ faire du sport<br><br>And 5 other symbols [3]
Quelle activité avez-vous faite l'après-midi? (choix multiple)	MULTI-SELECT `afternoon_activity`<br>01 ☐ travaillant à la ferme<br>02 ☐ cuisine<br>03 ☐ vendre des produits sur le marché<br>04 ☐ élevage d'animaux<br>05 ☐ prendre soin des enfants<br>06 ☐ prendre soin de son mari / femme<br>07 ☐ prendre soin des membres de la famille<br>08 ☐ va à l'école<br>09 ☐ chercher de l'eau dans le puits<br>10 ☐ récolte<br>11 ☐ travailler sur le terrain<br>12 ☐ je me reposais<br>13 ☐ regarder la télévision<br>14 ☐ aller au bar<br>15 ☐ aller au restaurant<br>16 ☐ faire du sport<br><br>And 5 other symbols [3]

Quelle activité as-tu pratiquée le soir? (choix multiple)	MULTI-SELECT evening_activity<br>01 ☐ travaillant à la ferme<br>02 ☐ cuisine<br>03 ☐ vendre des produits sur le marché<br>04 ☐ élevage d'animaux<br>05 ☐ prendre soin des enfants<br>06 ☐ prendre soin de son mari / femme<br>07 ☐ prendre soin des membres de la famille<br>08 ☐ va à l'école<br>09 ☐ chercher de l'eau dans le puits<br>10 ☐ récolte<br>11 ☐ travailler sur le terrain<br>12 ☐ je me reposais<br>13 ☐ regarder la télévision<br>14 ☐ aller au bar<br>15 ☐ aller au restaurant<br>16 ☐ faire du sport<br><br>And 5 other symbols [3]
Quelle activité as-tu pratiquée la nuit? (choix multiple)	MULTI-SELECT night_activity<br>01 ☐ travaillant à la ferme<br>02 ☐ cuisine<br>03 ☐ vendre des produits sur le marché<br>04 ☐ élevage d'animaux<br>05 ☐ prendre soin des enfants<br>06 ☐ prendre soin de son mari / femme<br>07 ☐ prendre soin des membres de la famille<br>08 ☐ va à l'école<br>09 ☐ chercher de l'eau dans le puits<br>10 ☐ récolte<br>11 ☐ travailler sur le terrain<br>12 ☐ je me reposais<br>13 ☐ regarder la télévision<br>14 ☐ aller au bar<br>15 ☐ aller au restaurant<br>16 ☐ faire du sport<br><br>And 5 other symbols [3]
Veuillez ajouter l'activité (ainsi que l'heure de la journée) et décrire, si possible	TEXT comment_activity<br>..

APPENDIX A — CATEGORIES

[1] Categories_LikertScale
Categories: 1:Tout à fait d'accord, 2:D'accord, 3:Ni en désaccord ni d'accord, 4:Pas d'accord, 5:Pas du tout d'accord

[2] Categories_Sensors
Categories: 1:Withings Pulse HR (noir poignet), 2:Tucky thermomètre (blanc tache), 3:Aucune

[3] Categories_Activity_Diary
Categories: 1:travaillant à la ferme, 2:cuisine, 3:vendre des produits sur le marché, 4:élevage d'animaux, 5:prendre soin des enfants, 6:prendre soin de son mari / femme, 7:prendre soin des membres de la famille, 8:va à l'école, 9:chercher de l'eau dans le puits, 10:récolte, 11:travailler sur le terrain, 12:je me reposais, 13:regarder la télévision, 14:aller au bar, 15:aller au restaurant, 16:faire du sport, 17:conduire un véhicule, 18:travaux ménagers, 19:j'ai fait du shopping, 20:nettoyer, 21:travailler assis

[4] nom_village: Nom du village du participant
Categories: 1:Barakuy, 2:Toni, 3:Biron Bobo, 4:Biron Marka, 5:Boron, 6:Bouni/Boune, 7:Bourasso, 8:Cisse, 9:Dankoumana, 10:Dembelela, 11:Denissa, 12:Denissa Mossi, 13:Diamasso, 14:Dionkongo, 15:Dina, 16:Dokoura, 17:Goni, 18:Kamadena, 19:Kemena, 20:Kodougou, 21:Koro, 22:Labarani, 23:Lei, 24:Lekuy, 25:Lemini, 26:Nokuy, 27:Ouette, 28:Pa, 29:Sampopo, 30:Seriba, 31:Sien, 32:Sikoro, 33:Sobon, 34:Solimana, 35:Sirakoro/Sirakorosso, 36:Tebere, 37:Tonsere, 38:Zanakuy, 39:Tissi, 40:Dara, 41:Bankoumani1, 43:Babekolon, 44:Bagala, 45:Biron badala, 46:Bissau, 47:Bokuy, 48:Damandigui, 49:Hinkuy, 50:Kamiankoro, 51:Kansara, 52:Kerena, 53:Konkuini, 54:Koredougou, 55:Moinsi, 56:Mourdie, 57:Sere, 58:Soin, 59:Tonkoroni, 60:BABAKUY, 62:BANGASSI-KORO, 63:BANGASSI_KOUROU, 64:BARANI, 65:NOUNA, 66:BOGO3, 67:BOULEMPORO, 68:BOULE, 69:DIAMAHOUN, 70:DIENWELY, 71:DJALLO, 72:DOURE, 73:GNIMANOU, 74:ILLA, 75:KAMANDADOUGOU, 76:KAREKUY3, 77:KESSEKUY, 78:KINSERE, 79:KOLONKAN GOURE BA, 80:KOLONKAN GOURE DIALLO, 81:KONKORO, 82:KORONI, 83:KOUBE, 84:KOULEROU, 85:MANEKUY, 86:MANTAMOU, 87:MEDOUGOU, 88:NABASSO, 89:NIAKO, 91:OUEMBOYE, 92:OUERESSE, 93:PAMPAKUY, 94:SEKUY, 95:SEKUY-IRA, 96:SOKOURA3, 97:SOUDOGO, 98:TIRA, 99:TOROKOTO, 100:WARIBERE, 101:YALANKORO, 102:BANAKORO, 103:BOGO4, 104:BOMBOROKUY, 105:BOREKUY, 106:DANEKUY, 107:GOMBELE, 108:KOMONKUY, 109:MARIASSO, 110:NIANKOUINI, 111:SADIGAN, 112:SAKO, 113:SOUANKUY, 114:TIRAKUY, 115:YABANA, 116:YALLO, 117:BA, 118:BANANA, 119:BANKOUMANA, 120:BARA, 121:BERKOUE, 122:BIDA, 123:BOKORO, 124:BONOUA, 125:BOUAKUY, 126:BOURIO, 127:DIEKUINI, 128:DIENA, 129:DIEKAN, 130:DJIBASSO, 131:DONKORO, 132:FONI-BORONKIN, 133:GNIMINI, 134:IRA, 135:KANSARA, 136:KIENEKUY, 137:KIRA, 138:KIE, 139:KOLONKAN, 140:KOLONKANI-SIRAKORO, 141:KOLONZO5, 142:KOMBORI5, 143:MANDARA, 144:MAOULENA, 145:MASSAKUY, 146:MOUNA, 147:NAIRENA, 148:OUAROKUY, 149:OURA, 150:OUROUKO, 151:PARAKUY, 152:PARANZO, 153:PIA N 1, 154:SABA, 155:SADIGNAKONO, 156:SAKUY, 157:SAMEKUY, 158:SARAKORO, 159:SENOULO, 160:SIEDOUGOU, 161:SOUMOUKUY, 162:SOUNE, 163:SOYE, 164:TIEME, 165:VORO, 166:AYOUBAKOLON, 167:BONIKUY, 168:DAR-ES-SALAM, 169:DASSI, 170:DOKUY, 171:DOUBALE, 172:GASSINGO, 173:ILABEKOLON, 174:KANADOUGOU, 175:KARASSO, 176:KEMENSO, 177:KENEKUY, 178:KOLONIDARA, 179:KOLONKOURA6, 180:MAKUY, 181:NEREKO, 182:SOKOURA6, 183:SOUM, 184:SOUMAKORO, 185:TOMIKORONI, 186:BAMPERLA, 187:BANGASSI-BOBO, 188:BANGASSI-ILLA, 189:BANGASSI-MAMOUDOUD, 190:BANKUY, 191:BASSAM, 192:BOANEKUY, 193:BOKUY7, 194:BOUKUY, 195:DAKUY, 196:DOUMBALA, 197:HENLEKUY, 198:KAREKUY7, 199:KIMBA, 200:KINI-KINI, 201:KOA, 202:KODARA, 203:KOLONZO7, 204:KONKUY-BOHO, 205:KONKUY-KORO, 206:KOURKUY, 207:LANFIERA, 208:MONTIONKUY, 209:MOUNAKORO, 210:NIAN, 211:POROKUY, 212:SAINT-CAMILLE, 213:SAINT-MARTIN, 214:SAINT-PAUL, 215:SAWOROKUY, 216:SIMBORA, 217:TENI, 218:TENI-PEULH, 219:TIOURKUY, 220:WANZAN, 221:ZEKUY, 222:ABAYE, 223:AOUREMA8, 224:BA-PEULH, 225:DAGA, 226:GANI, 227:KOLONKANI-BA, 228:KOMBORI8, 229:KONNA, 230:LONANI, 231:MAGADIAN, 232:OUORI, 233:SANAKADOUGOU, 234:SASSAMBARI, 235:SIEKORO, 236:SIEWALI, 237:SIGUIDE, 238:YARAN, 239:BANKOUMANI, 240:BOKUY9, 241:DINA, 242:KIKO, 243:KOLOKAN, 244:MADOUBA, 245:PIA N 2, 246:PORO, 247:TOUBA, 248:YOUROUNA, 249:AOUREMA1, 250:BARE, 251:DANTIERA, 252:DEMBO, 253:DIGANI, 254:DIONDOUGOU, 255:FARAKUY, 256:KAKI, 257:KALFADOUGOU, 258:KAREKUY1, 260:KOMBARA, 261:KONANKOIRA, 262:KONONIBA, 263:MANI, 264:PATIARAKUY, 265:SAINT-JEAN, 266:SAINT-LOUIS, 267:SIMBADOUGOU, 268:SOA, 269:SOKORO, 270:TENOU, 271:THIA, 272:TOMBODOUGOU, 273:ZOUN, 275:BANTOMBO, 276:BOTTE, 277:KALLE, 278:KOURY, 279:LANFIERA-KOURA, 280:SIELA, 281:SONO, 282:SORO, 283:ZAMPANA, 285:sontorokuy, 286:yevedougou, 288:Homokuy, 289:Daborokuy, 290:Warkuy, 291:Koncoba, 292:Sayokuy, 293:Poe, 295:Werimbere, 296:Noumbere

LEGEND

Legend and structure of information in this file

Name of section

Enabling condition for this section

Question title

Type of question, scope

Variable name

Answer options

SECTION 5: OTHER INCOME SOURCES

E s4_other_sources_which.Contains(98)

Duis aute irure dolor in reprehenderit in voluptate velit esse cillum dolore eu fugiat nulla pariatur?

MULTI-SELECT
SCOPE: PREFILLED

s4_rel_leaders_other

I This refers to family relations
E s3_time_other > 0
V1 s4_rel_leaders_which.Contains(98)
M1 Can not be itself
V2 (s3_time_other_breeding_advice <= (50 - s3_time_art_insem_advice)) ||s3_time_other_breeding_advice == 0
M2 This person is not in the list
F optioncode != s5_ignored_option_code

01 ☐ Community animal health workers
02 ☐ Private
03 ☐ Government
04 ☐ Livestock keepers association
05 ☐ NGO

And 5 other [13]

Additional information:
"I" — Question instruction
"E" — Enabling condition
"V1" — Validation condition №1
"M1" — Message for validation №1
"F" — Filter in Categorical questions

Link to full set in appendix

Breadcrumbs

Type or roster

Roster Title

CHAPTER 3 IDENTIFICATION /
Roster: LEADER RELATION DETAILS
generated by fixed list:

01 Ward Livestock Officer
02 Village Livestock Officer
99 Other (specify)

List items
